# Tissue-based Alzheimer gene expression markers–comparison of multiple machine learning approaches and investigation of redundancy in small biomarker sets

**DOI:** 10.1186/1471-2105-13-266

**Published:** 2012-10-15

**Authors:** Lena Scheubert, Mitja Luštrek, Rainer Schmidt, Dirk Repsilber, Georg Fuellen

**Affiliations:** 1 Institute of Computer Science, University of Osnabrück, Albrechtstr. 28, 49076 Osnabrück, Germany; 2 Department of Intelligent Systems, Jožef Stefan Institute, Jamova cesta 39, 1000 Ljubljana, Slovenia; 3 Institute for Biostatistics and Informatics in Medicine and Ageing Research, University of Rostock, Ernst-Heydemann-Str. 8, 18057 Rostock, Germany; 4 Leibniz Institute for Farm Animal Biology (FBN Dummerstorf), , Wilhelm-Stahl Allee 2, 18196 Dummerstorf, Germany; 5 DZNE, German Center for Neurodegenerative Disorders, Gehlsheimer Strasse 20, 18147 Rostock, Germany

## Abstract

**Background:**

Alzheimer’s disease has been known for more than 100 years and the underlying molecular mechanisms are not yet completely understood. The identification of genes involved in the processes in Alzheimer affected brain is an important step towards such an understanding. Genes differentially expressed in diseased and healthy brains are promising candidates.

**Results:**

Based on microarray data we identify potential biomarkers as well as biomarker combinations using three feature selection methods: information gain, mean decrease accuracy of random forest and a wrapper of genetic algorithm and support vector machine (GA/SVM). Information gain and random forest are two commonly used methods. We compare their output to the results obtained from GA/SVM. GA/SVM is rarely used for the analysis of microarray data, but it is able to identify genes capable of classifying tissues into different classes at least as well as the two reference methods.

**Conclusion:**

Compared to the other methods, GA/SVM has the advantage of finding small, less redundant sets of genes that, in combination, show superior classification characteristics. The biological significance of the genes and gene pairs is discussed.

## Background

Sporadic Alzheimer’s disease [1] is the most common form of dementia. It is an irreversible, neurodegenerative brain disease featuring clinical symptoms usually starting at an age over 65 years, although the early-onset Alzheimer’s disease, a rare form, can occur much earlier.

Even though there are a lot of studies on Alzheimer’s diseases, its causes and progression are not well understood. A full appreciation of the underlying molecular mechanisms could be the key to its successful treatment. In particular, identifying genes that have a different property in disease affected versus healthy tissues (biomarkers) could help both understanding the causes of the disease as well as suggest treatment options.

Based on gene expression data from different brain regions of patients diagnosed with Alzheimer’s disease and a healthy control group [2], we analyze the utility of identifying biomarkers by a wrapper approach involving a genetic algorithm and a support vector machine [3]. The same method showed good results selecting biomarkers for the pluripotency of cells [4]. In this paper, we will compare some of the results obtained for pluripotency to the results obtained for Alzheimer. While finalizing this comparison, we noted an inadvertent problem in the data processing in [4], leading to slightly elevated accuracies due to an incorrect handling of replicates. In this paper, all results reported for the pluripotency data set were re-done, using the correct design regarding replicates (see Methods).

One of the important advantages of the wrapper of genetic algorithm and support vector machine (GA/SVM) as a method to identify biomarkers is the observation that it finds small gene sets that are good biomarkers in combination. In particular, we identify and describe pairs of genes that are much better suited for separating the diseased and the healthy samples, as compared to the single genes of such a pair.

Recent studies [5-8] have identified new candidate genes associated with Alzheimer’s disease. The candidate genes selected in [5,6] are based on the expansion of reference gene sets whose role in the disease is already well defined. In contrast, we provide a method that allows the identification of new candidate genes for Alzheimer from microarray data, without including any prior knowledge. Therefore, we are able to use gene sets and networks already associated with Alzheimer’s disease as a first independent validation for the biological relevance of our results. The approaches in [7,8] are closer to ours in that they also do not rely on prior knowledge. They use Independent Component Analysis [7] and Special Local Clustering [8], respectively, to transform gene expression data, and then select candidate genes in a relatively straightforward fashion. In contrast, we work directly with gene expression data, and use a more complex method of selecting candidate genes.

## Results

In [4], we introduced the GA/SVM algorithm that shows good results identifying pluripotency related genes using a pluripotency-related (PLURI) data set. As we use the same technique for analyzing the Alzheimer’s disease-associated (AD) data set, part of the Results section is the comparison of the results obtained on the two sets. (See Methods section for details on these data sets.) We then continue and analyze the specific synergistic performance of gene pairs proposed by the GA/SVM approach using the AD data set as well as the PLURI data set.

### Classification

As a first step of our analysis we look at the classification performance of five different classification methods, implemented as described in the Methods section. Table 1 shows the cross-validated classification accuracy for all five methods on the AD data set. We also provide the results we obtained on the PLURI data set already published in [4] for comparison, based on the corrected cross-validation scheme (see Methods).

**Table 1:** Accuracy of six classifiers

	**AD**	**PLURI**
Naive Bayes	81.4%	87.1%
C4.5 decision tree	78.9%	95.1%
Nearest neighbor	87.0%	96.5%
Random Forest	87.0%	97.2%
SVM + Gaussian kernel	85.7%	97.9%
SVM + linear kernel	91.9%	99.0%

The classification results of different methods on the two 1,000 gene data sets AD and PLURI. The classification accuracy is computed as average from a 3-fold cross-validation.

For both data sets the SVM with linear kernel shows the highest classification accuracy with 91.9% on the AD data set and 99.0% on the PLURI data set. The lowest accuracies are obtained by the C4.5 decision tree classifier and the Naive Bayes classifier with an accuracy smaller than 82% on AD and smaller than 96% on PLURI. The performance of the other three classifiers is in the intermediate range. We further observe a large difference between the two data sets. No matter what classification method we use, the observed cross-validation accuracy is much lower for AD than for PLURI. As SVM with linear kernel shows the best results on both data sets, we use this classifier for evaluating the quality of the genes selected with different feature selection methods in the next section. Because the SVM is part of one of our feature selection methods, we use a random forest classifier as well as SVM with Gaussian kernel to evaluate quality of classification.

### Feature selection

Feature selection is used in machine learning to select a subset of relevant features that improves classification. We use feature selection by information gain, random forest and the GA/SVM to find biomarkers with a potentially high importance in Alzheimer’s disease.

For feature selection on the AD data set, we use the same methods as for the PLURI data set [4]. In the following section we display all results for the AD data set and the PLURI data set next to each other to ease the comparison of the results.

#### Classification performance of selected genes

In order to give a clear statement about the quality of the potential biomarkers found by the three feature selection algorithms, we compare the test set accuracies taking a cross validation approach, where the genes selected on the training set are used for classification. Therefore, we split all samples into three subsets and prepare each fold as described in the Methods section. The resulting 1,000 genes of each fold are sorted by their importance based on applying information gain, random forest and the GA/SVM on two subsets as the training set, where the GA/SVM is run 200 times on each fold. Then we select the best 50, 40, 30, 20, 15, 10, 5, 3, 2 and 1 genes for each algorithm and each fold and utilize these to compute the classification performance using an SVM with Gaussian kernel and one with linear kernel as well as a random forest on the respective test sets. As classification accuracy for each method, the average accuracy over all three folds is used. For each of the three folds two subsets were used for feature selection. To test the quality of the selected genes we train a classifier on these two subsets using the gene expression values of these genes as input. The classification accuracy is determined by using the remaining subset for testing (for more details see Methods section). As we use the SVM within the genetic algorithm, the odds are that the SVM might favor the genes found by the GA/SVM, this is why we use random forest as well.

**Figure 1:**
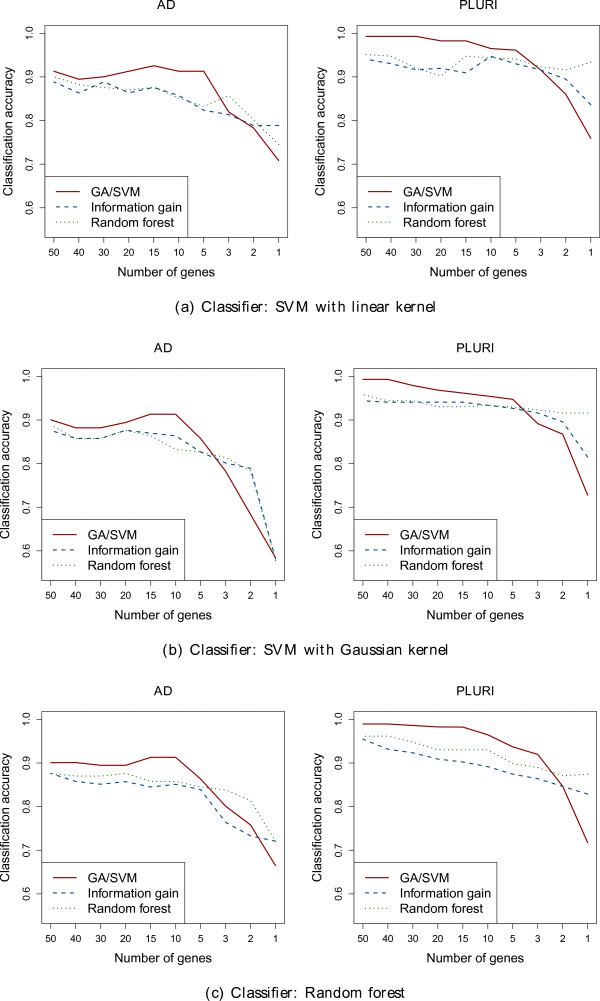
**Classification accuracy of selected genes.** Classification accuracy of three classifiers using incrementally smaller sets of genes, identified by our three feature selection methods.

In Figure 1, we plot the classification results. Using just a few biomarkers for classification, the genes selected by information gain and random forest are better suited for separating the samples into two classes, compared to the genes selected by the GA/SVM. However, taking 5 or more genes for classification, the genes selected by the GA/SVM perform better than the ones chosen by the other two methods. No matter if we use an SVM or random forest as classifier, we observe the same results.

#### Mutual information of selected genes

**Figure 2:**
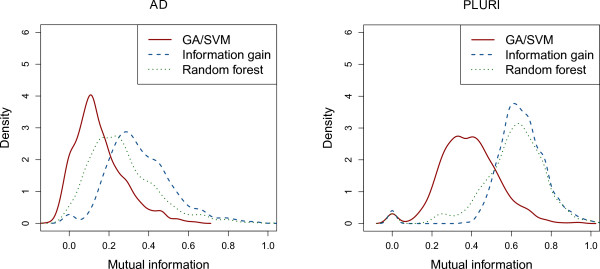
**Mutual information.** Density of mutual information of the top 50 genes for three feature selection methods.

For each feature selection method we construct all possible gene pairs from the 50 top-ranked genes. We calculate the mutual information [9] for each of those gene pairs. Figure 2 shows the Gaussian density estimations of the resulting mutual information values for the three feature selection methods.

For both data sets we find that the mutual information in the genes selected by our GA/SVM algorithm is lower than in the genes selected by information gain and random forest. Furthermore we observe a large difference between the PLURI and the AD data set. For all three methods the mutual information obtained in the top 50 ranked genes is higher in the PLURI than in the AD data set.

#### Performance of small biomarker sets

**Figure 3:**
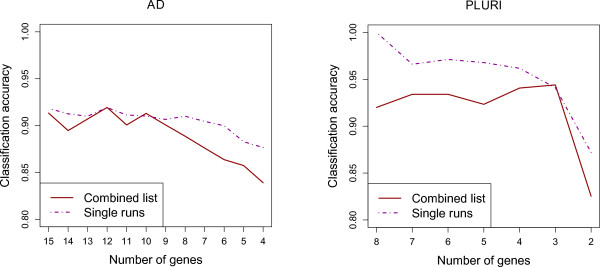
**Classification accuracy of small gene sets.** Classification accuracy measured by an SVM with Gaussian kernel. For training the classifiers, we use incrementally smaller sets of best ranked genes (combined list) and the small gene sets found by the GA/SVM in single runs.

Since the advantage of the GA/SVM is that it finds small sets of genes well suited for classifier training when used together, we compare the classification performance of the individual small gene sets selected in *single runs* of the GA/SVM to the performance of those genes found most often in all different runs (as before), collecting all genes together, from all runs of the GA/SVM. We use the same three folds as for the performance analysis described above, for selecting the genes for training and testing the classifier. As accuracy we calculate the average over all three folds. Again we run the GA/SVM 200 times on each fold and rank the genes for each fold by the frequency of their occurrence. Then we take incrementally smaller sets of the top ranked genes and use them for classification on the test set of the respective fold using an SVM with Gaussian kernel. Each of the runs of the GA/SVM results in a small set of genes which are supposed to work well together for classification. Thus, we use each set of genes for the training of a Gaussian SVM and combine the accuracies by computing the mean accuracies for all sets with a specific number of genes. This is done again for each fold separately and in a last step the average over all three folds is taken. Figure 3 shows the results of this comparison. The classification accuracy of the small sets found by the GA/SVM in a single run is usually higher than the accuracy of the combined list.

#### Top genes

Table 2 lists the top 20 most important genes selected by each of the three feature selection methods (GA/SVM, information gain and random forest) and a list of genes obtained by combining the results of the three methods as described in the Methods section. Here, we base the estimation of the important genes on all samples and increase the number of runs of the GA/SVM to 500.

**Table 2:** Top 20 genes

**GA/SVM**	**Information gain**	**Random forest**	**Overall**
loc642711	flj11903	**loc283345**	**loc283345**
prkxp1	tncrna	flj11903	**pcyox1l**
**loc283345**	loc283755	loc642711	**c6orf151**
sst	ptpn3	gprasp2	mid1ip1
ly6h	**pcyox1l**	tncrna	bcl6
ercc3	ppih	**pcyox1l**	ppih
loc643287	hsd17b7	mid1ip1	maff
tnni3k	gprasp2	pdzd11	sst
cdk2ap1	**loc283345**	maff	hsd17b7
fbxo16	**c6orf151**	loc283755	cdc37
gem	mettl7a	flj25477	ep300
taf3	mrps22	bcl6	flj11903
znf415	cdc37	palld	loc645352
loc285927	nfkbia	loc645352	prr11
mael	fam63a	eif3s12	taf3
supv3l1	rad51c	slc12a7	terf2ip
**c6orf151**	anp32b	mgc12488	scrib
fam54b	ubxd4	nfkbia	pdzd11
**pcyox1l**	terf2ip	**c6orf151**	gprasp2
ep300	bcl6	atp5b	mxi1a

The top 20 genes selected by the three feature selection methods. Additionally, an overall gene list of all three methods is shown. The genes occurring in all four lists are highlighted in bold.

We observe more similarities between the top genes identified by information gain and random forest. However, they both have genes in common with the genes found by the GA/SVM. Besides, the top three genes in the overall list are ranked among the top 20 by all three algorithms.

### Enrichment analysis

To demonstrate the biological relevance of our biomarker candidates we perform an over-representation analysis (as described in the Methods section). We study the over-representation of the genes identified as most important biomarkers by the three feature selection methods (information gain, random forest and GA/SVM) in pre-defined lists of genes known to play a role for Alzheimer’s disease. Our gene sets are analyzed for enrichment with respect to various gene sets associated with Alzheimer’s disease. Since there is no pre-defined number of most important genes, we start with the best 40 genes and increase the number in steps of 20 up to 200 genes. That way we obtain 9 gene sets for each of the three feature selection methods.

**Figure 4:**
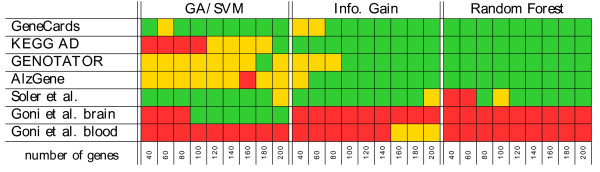
**Gene set enrichment analysis.** Results of the gene set enrichment analysis for incrementally larger sets of genes found by the three feature selection methods. Green: significant enrichment (p-value < 0.05). Yellow: enrichment. Red: no enrichment.

In Figure 4 we find a significant enrichment (p-value < 0.05) for nearly all of the tested gene sets in the list of genes associated with Alzheimer’s disease by GeneCards (http://www.genecards.org). The biomarkers found by information gain and random forest are significantly enriched in the KEGG pathway of Alzheimer’s disease [10] as well as in the two collections of Alzheimer’s disease related genes by Genotator [11] and AlzGene [12]; in case of GA/SVM, only enrichment (without significance) is observed. The genes found by the GA/SVM show a significant enrichment, however, in the brain specific gene lists of Soler et al. [13] and Goni et al. [14]. Notably, while the list of Soler et al. is also enriched in the genes found by information gain and random forest, the gene list of brain-specific tissue found by Goni et al. is only significantly enriched in the gene lists of size 100 and more that are derived by our GA/SVM. In the blood specific list of Goni et al. none of the three feature selection methods yields gene sets showing an enrichment.

### Interaction analysis

As shown in the previous sections, our GA/SVM finds genes that are suitable for separating two groups of samples on the basis of their gene expression values, but there are other methods showing a good performance on this problem as well. Compared to information gain [15] and random forest [16], the GA/SVM differs fundamentally, however. During each run it selects a specific small set of genes. On average, these sets show an even better classification capability than the genes of the combined list (see Figure 3). Explicitly selecting small sets of features, we can not only find good biomarkers, but we can also observe joint occurrences of genes well suited for training classifiers. Some gene pairs in the selected feature sets consist of two genes occurring at the frequency expected if we assume that the genes contribute independently to the classification performance of the SVM that is wrapped by the GA. Other gene pairs are present more frequently (over-represented) or less frequently (under-represented) than expected, so we assume that those genes are not independent of each other. These gene pairs are the subject in the analysis that follows.

As described in the Methods section, for each gene pair we calculate the strength of over- or under-representation (*importance_jo*) considering 3,000 small gene sets selected by the GA/SVM. This way, we are able to label which gene pairs are most over-represented and which are most under-represented in the small gene sets. We use 3,000 small sets to ensure a sufficiently large number of significantly over- and under-represented gene pairs for our analysis, where significance is defined as described in the Methods section. For the same reason, we examine gene pairs instead of triples or combinations of more than three genes. Further, we use an SVM with Gaussian kernel to determine the SVM classification accuracy (*SVMacc*), the mean gain of accuracy (*SVMgainMean*) and the minimal gain of accuracy (*SVMgainMin*) of each gene pair (see Methods section).

**Figure 5:**
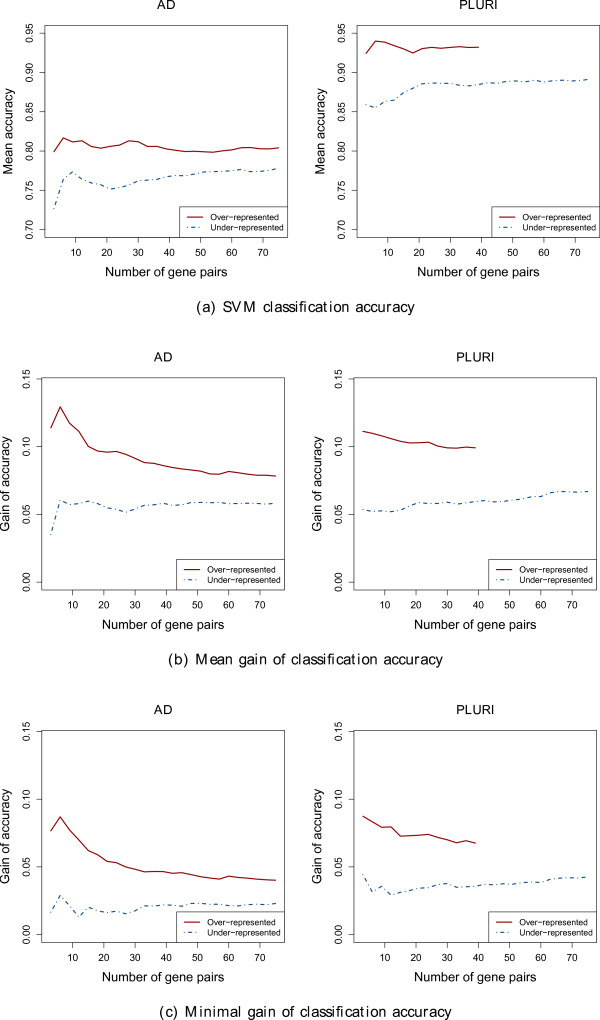
**Accuracy and accuracy gain of the most over- and under-represented gene pairs.** Accuracy and accuracy gain of the most over- and under-represented gene pairs. As we obtain only 38 significantly over-represented gene pairs for the PLURI data set, the upper lines for the PLURI data set are truncated.

Figure 5 shows the SVM classification accuracy (panel(a)), the mean gain of accuracy (panel(b)) and the minimal gain of accuracy (panel(c)), each time averaged over the 3, 6, 9, …, 75 most over- and under-represented gene pairs.

In Figure 5(a) we consistently obtain a higher classification accuracy for the most over-represented gene pairs than for the most under-represented gene pairs. However, we are not primarily interested in the absolute classification accuracy of those gene pairs, but in the relative gain of accuracy observed by combining two genes. In Figures 5(b) and 5(c) we illustrate the gain of accuracy we obtain by combining two genes. The mean accuracy gain as well as the minimal accuracy gain is significantly larger for the most over-represented gene pairs than for the most under-represented gene pairs (two-tailed t-test [17], p-value ≤ 0.05). Furthermore we observe a decrease of accuracy gain, the more less important over-represented gene pairs we add. The inverse applies for the under-represented gene pairs. Here we see a slight increase of accuracy gain, the more under-represented gene pairs we add.

**Figure 6:**
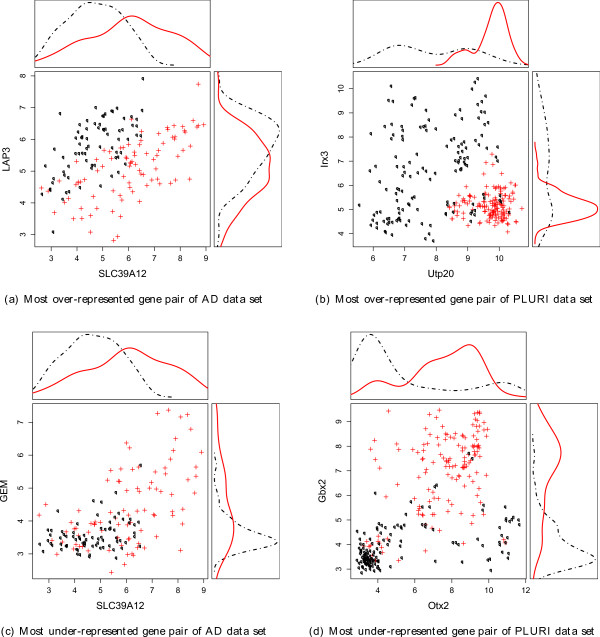
**Visualisation of the most over- and under-represented gene pairs.** Visualisation of the most over- and under-represented gene pairs of the AD and the PLURI data set. The scatter plots show the distribution of the gene expression values of the samples for the pairs of genes. The density diagrams show the distribution of gene expression values of the samples for single genes. The pluripotent and Alzheimer affected samples are marked by + and continuous lines (red), the non-pluripotent and healthy samples are marked by 0 and dotted lines (black).

Figure 6 shows the gene expression diagrams of the single most over- as well as the single most under-represented gene pair for each data set. The figure gives a visual impression of the accuracy gain obtained by combining two such single genes, enabling inspection of their expression patterns in two dimensions. The line charts show the Gaussian kernel estimates of the gene expression distribution of the single genes. The distribution is shown for each of the two categories of samples separately, using the red line for affected (Alzheimer respectively pluripotent) and the black dotted line for non-affected samples (healthy respectively non-pluripotent). The scatterplot shows the expression values of both genes in two dimensions. In the density charts of the most over-represented gene pairs we see a large overlap of the two classes, whereas the overlap in the scatterplot is much lower. For the most under-represented gene pairs we see a large overlap of the two classes in the density diagram as well as in the scatterplot. The corresponding SVM accuracy of the single genes as well as the SVM accuracy for the gene pairs and the mean and minimal gain of accuracy are given in Tables 3 and 4. We note that the accuracy gain is high for most over-represented gene pairs.

**Table 3:** Most over/under-represented gene pairs

**Gene pair**	***SVMacc***	***SVMgainMean***	***SVMgainMin***	**Data set**
SLC39A12/LAP3(*↑*)	80%	14%	10%	AD
SLC39A12/GEM(*↓*)	71%	2%	1%	AD
Utp20/Irx3(*↑*)	92%	13%	11%	PLURI
Otx2/Gbx2(*↓*)	88%	4%	3%	PLURI

Most over(*↑*)- and most under(*↓*)-represented gene pairs and their corresponding accuracies.

**Table 4:** Single genes of the most over/under-represented gene pairs

**Gene**	***SVMacc***	**Data set**
SLC39A12	70%	AD
LAP3	63%	AD
GEM	69%	AD
Utp20	81%	PLURI
Irx3	77%	PLURI
Otx2	83%	PLURI
Gbx2	85%	PLURI

Accuracies of the single genes contained in the most over(*↑*)- and most under(*↓*)-represented gene pairs (see Table 3).

## Discussion

We split the discussion into three parts. The first part concerns the quality of the three feature selection methods information gain, random forest and GA/SVM as well as the classification performance of different classifiers. The second part deals with the over- and under-represented gene pairs selected by the GA/SVM. Finally, we discuss the biological relevance of the selected genes.

### Comparison of the three feature selection methods

Usually, regulatory processes in a cell are very complex and single genes are not able to explain all aspects of a biological cell state. For this reason, combining the best-ranked genes to improve the classification capability is a frequently used approach [18,19]. However, combining *redundant* genes usually does not improve the classification capability of a gene set much.

Figure 1 shows the classification capability of incrementally smaller sets of genes ranked top by our three feature selection methods. Independently of the classifier used to evaluate the resulting gene list, we observe very similar results. There does not seem to be any particular preference for the GA/SVM if the SVM is used to measure performance. This suggests that the selected genes generally have a good classification capability. Comparing classifiers only trained with the best 50 genes (Figure 1) to classifiers trained with the larger set of 1000 genes (Table 1), we find nearly no difference in classification accuracy. We assume this to be another piece of evidence for the quality of the selected biomarkers.

For both data sets we observe the same two main points. First, if we use only few genes for training a classifier, the genes selected by information gain and random forest show better classification results than the genes selected by GA/SVM. Second, if we use five or more top ranked genes, the genes selected by GA/SVM are better.

Expanding on [4], we explain these observations by the differences between the three feature selection methods. Information gain ranks a gene only considering the gene expression values of this single gene. For this reason we expect a lot of redundancy among the top ranked genes. The results in Figure 1 show that the accuracy increases slowly as we increase the number of genes used for classification, which probably happens precisely because combining redundant genes does not improve the classification accuracy much. Nevertheless, the Shannon entropy [20] (used by information gain) seems to be a good ranking criterion, as the top ranked gene shows a very good classification capability.

In contrast to information gain, random forest determines the importance of a gene based on the classification capability of that gene in multiple trees. This way the score of each gene depends on the gene expression value of other genes as well. Nevertheless, usually the list of top ranked genes still contains many redundancies (see Figure 2). This explains why combining multiple top ranked genes does not distinctly improve the classification capability of the trained classifier.

In contrast to these two methods, our GA/SVM has a strong tendency to eliminate redundant genes. This is demonstrated by the observed low values of mutual information (see Figure 2) measuring the mutual dependency of gene pairs. Thus we hypothesize that each small gene set, selected by the GA/SVM in a single run, consists of genes that fulfill different functions for the specific biological state. If multiple genes are redundant, only one of those redundant genes tends to be chosen by chance, as a member of a gene set. For this reason, ranking the genes by their frequency of occurrence in the small gene sets, genes with many redundant partners are infrequent. The genes best ranked by the GA/SVM tend to have no redundant partners in the list of top ranked genes concerning the specific biological state (Alzheimer affected; pluripotent). Combining top ranked genes strongly improves the classification capability of a gene set, as can be seen in the sharp rise (from right to left) in the accuracy as the number of genes increases in Figure 1.

Analyzing the 50 top-ranked genes of each feature selection algorithm we observe that the genes selected by the GA/SVM algorithm on average show less pairwise mutual information than those selected by information gain and random forest. As the mutual information of two genes is a measure for the mutual dependency of the genes we assume that on average two genes selected by our GA/SVM algorithm depend on each other in a weaker degree than genes selected by the other two feature selection methods. As a high mutual dependency reveals redundancy between the genes, this supports our assumption that the gene lists selected by the GA/SVM algorithm contain less redundancies than the genes selected by information gain and random forest.

Even though GA/SVM is known to show good results in feature selection [3,4,21,22] the utility of the small gene sets selected during a single run has not been investigated in-depth yet. In the following section we consider this topic.

### Analysis of the small gene sets of GA/SVM

Many machine learning methods used for learning biomarkers, such as information gain [15] and random forest [16], perform only univariate ranking of genes. Although the top-ranked genes are valuable hints for understanding the underlying molecular mechanism of cellular processes, these processes are usually more complicated than single genes are able to explain. We are, therefore, interested in small sets of genes that best distinguish Alzheimer diseased versus healthy tissues (respectively pluripotent versus non-pluripotent cells).

In the previous section we discussed that small sets of genes obtained from the GA/SVM combined list are better suited for classification than those from information gain and random forest. Small sets individually selected by the GA/SVM have an even higher classification accuracy, as seen in Figure 3. For this reason, we conclude that the assembly of specific genes in a small set plays an important role for the prediction accuracy of classifiers using the genes selected by our GA/SVM. Therefore, it is useful to examine gene pairs that occur more frequently or less frequently than expected in the small gene sets of the GA/SVM.

In Figure 5 we display the difference between the most over- and under-represented gene pairs in absolute classification accuracy as well as in mean and minimal gain of accuracy. We observe that gene pairs often selected together in a single small gene set of the GA/SVM are on average better suited for separating the two groups of samples than those gene pairs rarely selected together (Figure 5(a)). Further, we observe that the GA/SVM prefers to assemble those genes whose combination leads to an increase (gain) of classification accuracy (Figure 5(b) and Figure 5(c)). We propose that non-redundant genes are chosen together much more often than redundant genes.

Figure 6 shows the most over-represented and under-represented gene pair for both data sets. The corresponding SVM accuracies can be found in Tables 3 and 4. For the AD data set the two genes LAP3 and SLC39A12 individually have a low classification capability (accuracy: 63% and 70%, respectively). However, LAP3/SLC39A12 is the most over-represented gene pair in the AD data set. Combining the two genes increases classification accuracy to 80%. Figure 6(a) illustrates the similar distribution of the two sample groups regarding the single genes, as well as the straight separability of the samples using both genes. Using a simplified rule, we may classify a sample as Alzheimer-affected if the gene expression value of SLC39A12 is larger than the expression value of LAP3.

For the most over-represented gene pair of the PLURI data set we make some similar observations. Combining the single genes Irx3 and Utp20 with individual classification accuracies of 77% and 81% increases accuracy to 92%. In Figure 6(b) we find the sample distribution concerning the single genes even better separable than for Lap3/SLC39A12 (see Figure 6(a)). Using a simple rule, we classify a sample as pluripotent if Utp20≥8 and Irx3≤6.

For the two most under-represented gene pairs in Figure 6(c) and Figure 6(d) we cannot determine such an easy rule for distinguishing the two sample groups. From a biological point of view we hypothesize that the genes contained in the pairs occurring in the small sets selected by the GA/SVM more often than expected are responsible or indicating the specific biological state, but we assume that they are not co-regulated and, therefore, not correlated. Instead we hypothesize that the genes fulfill different functions with respect to the specific biological state. We find indirect evidence for this hypothesis by inspecting Gbx2/Otx2, which constitute the most under-represented gene pair in the PLURI data set. Otx2 and Gbx2 are both known to play a role in pluripotent and undifferentiated stem cells [23,24]. Further, Gbx2 and Otx2 interplay as antagonists in cellular processes [25-27]. This negative correlation suggest that the two genes are redundant and, therefore, selected together rarely in a small set of genes by the GA/SVM. Inspecting the other gene pairs displayed in Figure 6 we find no reference to their interactions or redundancy in the literature. Thus, it is future work to investigate our hypothesis in more detail, optimally in a systematic way.

### Comparison of the two data sets

Performing the same analyses on two different data sets (PLURI and AD) enables us to compare the two data sets with each other and draw some conclusions about the two biological phenomena.

Besides many similarities between the two data sets already discussed in the two previous sections we also observe an important difference. Independently of the analysis performed, we observe a difference in absolute classification accuracy between the two data sets. Performing the same analysis, the accuracy obtained on the PLURI data set is usually at least 5% higher than on the AD data set (see Figures 1, 3 and 5(a) as well as Tables 1, 3 and 4).

Another interesting point is the different size of the small gene sets selected by the GA/SVM on AD and PLURI. Starting with approximately 15 genes in the start chromosomes of the GA/SVM, the algorithm selects up to 15 genes in the final small sets on the AD data set but only up to 8 genes on PLURI. For that reason we can assume that more genes are required for separating Alzheimer’s disease affected samples from healthy ones than pluripotent from non-pluripotent.

There are two possible reasons for the differences between the two data sets. (1) As the size of the two data sets differs a lot (containing 286 samples in the PLURI and 161 samples in the AD data set) and machine learning methods work best on large data sets, increasing the number of samples of AD could improve the ability of training a good classifier. (2) There are differences in the number of genes involved in pluripotency and Alzheimer’s disease, and in the way these genes function together. This could lead to specific difficulties in the classification problem based on the respective data sets. Both reasons are also supported by the different sizes of the small sets selected by the GA/SVM.

### Biological relevance of the selected genes

In this subsection we discuss the biological relevance of our results on the AD data set.

First, we elaborate on the enrichment analysis performed on the best genes selected by the three feature selection methods (see Figure 4). Second, we discuss the top genes in detail (see Table 2).

The gene list provided by GeneCards (http://www.genecards.org) when searching for Alzheimer’s disease is the most extensive one. All of the gene sets computed by the three feature selection methods show a significant enrichment. For the two gene lists offered by Genotator [11] and AlzGene [12] the biomarkers selected by information gain as well as random forest show a significant enrichment. The genes selected by the GA/SVM still show an enrichment. The experimental gene expression array studies of Soler et al. [13] (brain) and Goni et al. [14] (blood+brain) show an interesting point. Whereas the genes found by the GA/SVM are enriched in both studies concerning the brain, for information gain and random forest we only find an enrichment in the gene list of Soler et al. As we use only samples of brain tissues, it is understandable that none of the methods show an enrichment in the blood-based genes found by Goni et al.

The top five genes we found using the GA/SVM are LOC642711, PRKXP1, LOC283345, SST and LY6H. Although some of these genes are not yet well characterized, we can identify a relevance for Alzheimer’s disease for the majority of them.

The GA/SVM wrapper method found that LOC642711 is a very good choice for a small set of genes that can discriminate Alzheimer-affected brain tissue from non-affected brain tissue. However, LOC642711 has a RefSeq status of ’withdrawn’. To shed some light on this, we performed a nucleotide BLAST search with the withdrawn RefSeq, accession XM_931285.1, using standard parameters. The best match (see Additional file 1) is NR_036650.1, the ’WAS protein homolog associated with actin, golgi membranes and microtubules pseudogene (LOC100288615)’, which is 100% identical to our probe, over 75% of its length. This pseudogene is abbreviated as WHAMMP3, WHAMML12 and WHDC1L1 (according to GeneCards), and very recently is was shown to be part of a duplication on chromosome 15 associated with Alzheimer [28]. Also, sorting in post-golgi compartments has been implicated in AD [29]. Further analyses and experiments are needed to find out about the expression pattern of this pseudogene and its possible involvement in Alzheimer’s disease.

Recently, PRKXP1, SST and TNCRNA (also known as NEAT1, listed second by information gain) were identified by Squillario and Barla [30] as part of a 39 gene signature implicated in Alzheimer’s disease.

LOC283345, known as RPL13P5, is ranked by all three feature selection methods as one of the best 20 genes, although we have not found any link to Alzheimer’s disease. However, RPL13 has been implicated in severe Alzheimer [7] although it is also known as a housekeeping gene, in qRT-PCR studies in autopsy brain tissue samples from control and Alzheimer diseased cases [31] and it indeed shows a very low log fold change of 0.04 in our data set. However, its pseudogene 5 shows a very high log fold change of 0.96. Therefore, we assume that there is an unknown link between the pseudogene and Alzheimer’s disease, possible mediated by the original RPL13 gene. Alternatively, there may be unknown phenomena that confound the distinction between the pseudogene and the gene itself.

PRKXP1 is another pseudogene; interestingly the original gene PRKX is patented as an Alzheimer’s disease diagnostic and therapeutic target (http://www.google.com/patents/US20090136504).

SST (somatostatin), see also [30], expression was shown to be decreased in cortex and hippocampus of Alzheimer-affected brains [32]. SST also occurs in the top 20 overall list, since it is ranked high by information gain and random forest as well.

LY6H is patented as a brain-specific gene for treating Alzheimer’s disease (http://www.freepatentsonline.com/y2004/0254340.html).

Besides LOC283345, LOC642711 and TNCRNA (see above), the random forest ranks FLJ11903 and GPRASP2best. The function of the pseudogene FLJ11903 is not yet known. GPRASP2 encodes a protein that was shown to interact with several GPCRs (G protein-coupled receptors), which are relevant for the signal transduction system in Alzheimer’s disease [33,34].

Similar to random forest, information gain ranks FLJ11903 and TNCRNA best. It further chooses LOC283755, PTPN3 and PCYOX1L as most important genes. The protein encoded by PTPN3 (also known as PTPH1) belongs to a family known as cellular process regulating signaling molecules. A PTPH1 inhibitor is patented for the treatment of Alzheimer’s disease (http://www.freepatentsonline.com/y2011/0015254.html). Moreover, PTPN3 is a phosphatase and phosphorylation of the tau protein is considered highly relevant for AD progression [35]. The two genes LOC283755, also called HERC2P3, and PCYOX1L are not yet related to Alzheimer’s disease. Nevertheless, PCYOX1L is the second highest ranked gene in the overall list.

An interesting gene in the overall list, following LOC283345 and PCYOX1L, is C6ORF151. As the two others, it is ranked by all three selection methods as one of the top 20 genes. Likely, C6ORF151 is involved in U12-type 5’ splice site recognition; also known as SNRNP48 it participates in the massive transcriptional downregulation seen at late stage neurodegenerative (ALS) disease affecting mRNA metabolism and processing as well as RNA splicing [36].

The gene ranked 4th overall, MID1IP1, is among the top-10 genes found upregulated in the Alzheimer neocortex [37]. Finally, the gene ranked 5th overall, BCL6, is (together with CD24) the only immunity-related gene with significantly higher expression in severe Alzheimer’s disease that was singled out by principal component analysis (PCA) [7].

Recapitulating, we can demonstrate that the majority of the genes found by the three feature selection methods are related to Alzheimer’s disease. The genes not yet associated with Alzheimer’s disease will have to be further examined.

## Conclusion

Using our GA/SVM wrapper approach we identified new candidate biomarkers and, moreover, small sets of these, for Alzheimer’s disease, potentially providing new insights into the associated molecular processes. We expect that future biological experiments can test some of our computational predictions.

Compared with two popular feature selection methods, information gain and random forest, our GA/SVM shows the most promising results in finding new potential biomarkers. Usually, single genes are not responsible for the behavior of a cell. That is why we are also interested in finding genes who together are best suited to distinguish Alzheimer-affected cells from healthy ones, as compared to the single genes. While common feature selection methods do not consider such gene interactions for finding biomarkers, our GA/SVM not only considers these interactions in biomarker selection, it also enables us to identify pairs of genes relevant for Alzheimer’s disease that classify the samples very well when they are used in combination.

One promising way to improve the GA/SVM approach that we consider important future work is the use of specialized recombination and mutation operators based on directly feeding back ranking information based on the SVM results. Such approaches have been previously described in [38-40].

In the future, we would like to test our method on many more data sets, describing a wider variety of cellular phenotypes and diseases.

## Methods

### Gene expression data

On the subject of Alzheimer’s disease, surprisingly few large studies of gene expression exist. We use the data set GSE5281 as a comparatively large data set with raw data available. This data series consists of 161 samples of six different brain regions. 87 samples are of patients diagnosed with Alzheimer’s disease whereas the other 74 samples are from a healthy control group [2]. The experiments are based on an Affymetrix human genome U133 Plus 2.0 array. We refer to this data set as the AD data set.

As the samples of different brain regions partially originate from the same volunteers, we deal with partially correlated sample structures. To perform a proper cross validation on the data set, we ensure that all samples of the same patient are either in the training or in the test set. This way the training remains completely independent of the testing. Each sample is treated as a specific characteristic of either an affected or an unaffected brain and analyzed as if being an independent measurement. Furthermore, each sample can be associated with one of six brain regions. For each brain region the data set consists of approximately the same number of samples. To ensure that we identify genes that are usable for distinguishing between Alzheimer affected and non-affected samples and not between samples of different brain regions, we additionally take care of using uniform proportions of the brain regions in the training and test sets.

For comparison, we also discuss feature selection and machine learning results obtained with another data set referred to as PLURI. This set contains 146 pluripotent and 140 non-pluripotent samples of mouse cells. Here, all experiments are based on an Affymetrix mouse genome 430 2.0 array. The PLURI data set contains multiple replicates. To enable a proper cross validation with independent training and testing, in this paper we ensure that all replicates of a sample belong either to the training or the test set. We inadvertently did not follow this approach in [4], reporting slightly elevated accuracies there. A side-by-side comparison of all results for Alzheimer and pluripotency can be found in the Additional file 2.

### Data preprocessing

We accomplish data preprocessing in the same way as described in [4]. In the following, we give an overview of this process.

First, we summarize the Affymetrix gene expression arrays by using the Affymetrix Power Tool [41] implementing the robust multi-array average (RMA) method [42,43]. We use unmodified perfect match intensities for background adjustment, quantile normalized intensities [44] and median polish as summation method [42,43].

Before further filtering the data set, we combine all Affymetrix probe set identifiers that correspond to the same gene symbol from the UniGene record [45] by calculating the mean value. At this point, the two data sets contain gene expression data for 19,762 (AD) and 20,668 (PLURI) genes.

We split the analysis of the two data sets into two parts: (1) Analyzing the performance of classification algorithms and (2) finding best biomarkers. For (1) we use a suitable cross-validation approach with independent training and test sets. For that reason, we split the data sets into three subsets, to set up a three-fold cross-validation process using the same folds for each classification and feature selection algorithm. For each of the three folds two subsets are used for training, whereas the remaining subset is used for testing. The partitioning of the two data sets, following the rules set forth in the first section (’ Gene expression data’), can be found in the Additional files 3 and 4. In order to find the best biomarkers (2), we avoid information loss by using the whole data set for the feature selection process.

As the first step of our analysis, we perform the following filtering process for each of the three training sets (1) as well as for the whole data set (2), for both AD and PLURI. As we are interested in genes that are responsible for distinguishing the two classes of samples (affected and unaffected by Alzheimer’s disease, pluripotent and non-pluripotent), genes that show different expression values between these two groups of samples are identified by applying a two-sample t-test for samples with unequal variances. Then, the genes are sorted by their corrected p-values based on the concept of false discovery rate [46]. We dismiss all genes with a false discovery rate estimate larger than 0.1. After removal, we calculate the fold changes of the remaining genes. The fold change is computed using the difference between the means of the two groups of samples taking the normalized expression data at the log level. The genes are finally sorted by their fold change in decreasing order, and the data set used for feature selection consists of the first 1,000 genes in this ranking.

### Classification

We use six classification methods, all provided by the Weka machine learning suite [47], namely Naive Bayes [48,49], C4.5 decision trees [49,50], Random forest [16], Nearest neighbor [49,51] and SVM [49,52] with linear and Gaussian kernel.

As measurement of the classification performance of these algorithms we use their classification accuracies. The classification accuracy is defined as the number of correctly classified instances divided by the total number of instances.

All parameters of the machine learning algorithms are kept at Weka’s default values with two exceptions. First, for the SVM with Gaussian Kernel, we use the default parameters of LibSVM [53], because we use the LibSVM implementation inside the GA/SVM. Second, for classification with random forest, we build 1000 trees (default 10), to reduce variability of the results.

### Feature selection

We use a wrapper approach combining genetic algorithm and support vector machine (GA/SVM) [4], as well as two well known machine learning methods, information gain [15] and random forest [16]. As the GA/SVM approach enables us not only to find single biomarkers, but also to identify small sets of genes that are jointly best suited for training a classifier, this is the method we are most interested in.

#### Genetic algorithm

Feature selection with GA/SVM is performed using our own implementation described in detail in [4], briefly outlined in the following paragraphs.

The genetic algorithm [54] is a heuristic search method based on the processes of natural evolution. Starting with a population of chromosomes that encode problem solutions (in our case the selection of genes/biomarkers) it improves these chromosomes step by step. This process proceeds over numerous generations using recombination, mutation and selection until a stopping criterion is met, in our case 25 generations. The genetic algorithm is known for good results for a wide range of search problems.

We start with a population of 200 chromosomes, each describing a set of approximately 15 genes that are considered potential biomarkers, randomly chosen from the filtered list of 1000 genes. This gene set is scored by a fitness function based on the classification performance using a support vector machine [52], as follows:

$$\begin{array}{l} \text{fitness}=\text{(1-weight)}\cdot \text{accuracy + weight}\\ \;\times \frac{\text{numberOfAllGenes}-\text{numberOfSelectedGenes}}{\text{numberOfAllGenes}}. \end{array}$$

The first part of the fitness function ensures a preference for gene sets with a high classification performance, whereas the second part favours the selection of small gene sets. As our focus is on finding correct biomarkers we choose a small value for *weight*, setting it to 0.2 [55].

The term *accuracy* describes the classification accuracy of an SVM with Gaussian kernel. We use the LibSVM [53] implementation with default parameters performing a six-fold internal cross-validation to estimate the accuracy of a given gene set. Fitness also depends on *numberOfSelectedGenes*, the number of genes that the chromosome contains. In our case *numberOfAllGenes* is constantly set to 1,000 as we are working on data sets which each consist of 1,000 genes.

Beginning with the start population, we use recombination, mutation and selection to improve it. First, two chromosomes are chosen randomly depending on their fitness, using roulette wheel selection. Using uniform cross-over [56] a new chromosome is generated from the two parent chromosomes. This way 160 new chromosomes are generated. Furthermore, we perform a mutation on 20 more chromosomes, also chosen by roulette wheel selection. By mutation, we change approximately 0.15 percent of the bits (encoding the selection of genes). After performing cross-over and mutation we choose the best 200 chromosomes out of the 380 available as starting point for the next generation. This process is repeated 25 times.

The algorithm results in small sets of genes that are best suited for separating the samples into two classes. In order to assign a score to each gene and make the results comparable to random forest and information gain, we run the GA/SVM multiple times and count the frequency of occurrence of each gene in the resulting gene sets. For the cross-validated performance analysis we run the algorithm 200 times, while using the whole data set for feature selection we run it 500 times.

For the interaction analysis we run the GA/SVM 3,000 times and use the resulting small sets for analysis.

A java implementation of the algorithm, NewGa.jar, is available at http://sourceforge.net/projects/gasvmbmc/files/.

#### Random forest

The machine learning method random forest [16] is based on an ensemble of decision trees. Starting with a training set of *n* samples and *m* genes, a tree is built by selecting *n* samples of the training set randomly with replacement. Out of the *m* genes, *k* genes (with *k*<<*m*) are selected randomly. First, the algorithm chooses the gene with the largest entropy [20] as the root of the tree and splits the samples into two subsets based on the expression values of this gene. For each child node the algorithm again chooses the gene with the largest entropy and splits the remaining sample set. This process is repeated recursively until the leaves contain samples of one class only. As the random forest consists of several of such decision trees, in our case 1000, classification is performed by classifying a sample by all trees separately and determining the final class by majority voting.

Selecting the samples for growing a tree randomly with replacement, some samples are selected more than once and some are never selected. These never selected samples are called out-of-bag instances. To get a measure of the importance of a gene *g*, the out-of-bag instances are classified and the number of correct classifications are counted as *c*_*before*_. Before classifying the out-of-bag instances again, the values of gene *g* in the out-of-bag instances are permuted randomly. The new number of correctly classified instances is called *c*_*after*_. The importance of a gene *g* is given by the difference between *c*_*before*_and *c*_*after*_, averaged over all the trees in the forest, it is called mean decrease accuracy.

Feature selection using random forest is performed using a custom Weka distribution by Livingston [57].

#### Information gain

The information gain of a gene *g* quantifies the information one gains about a class *c* conditioned on knowing the gene expression value of *g*, neglecting the information of all other genes. Feature selection with the information gain is performed using Weka machine learning suite [47].

### Top genes

To obtain a list of the top 20 most promising biomarkers, the importance of each gene was computed by each of the three feature selection methods, the information gain [15], the random forest [16] and our GA/SVM [4].

The information gain and the random forest both assign a real-number importance score to each gene. While the information gain is a deterministic method, the scores computed by the random forest vary, depending on the randomly built trees. To minimize the variance, the importance score given by the random forest is averaged over three runs. The GA/SVM does not give an explicit score. It returns a small set of genes that is suitable for separating the given samples into two classes. To get suitable importance scores, we run the GA/SVM 500 times and count the occurrence of each gene, this count is used as the score.

These scores are comparable on the basis of ranking the genes with respect to their importance. To compute a list of genes combining the information of all three methods, the ranks (not the scores) for each gene are averaged over the three methods and the top 20 genes are chosen by their average rank.

### Gene set enrichment analysis

We apply a gene set enrichment analysis to evaluate the biological relevance of our results. To find out whether the genes we select in case of the AD data set are biologically relevant for Alzheimer’s disease, we compare the genes selected on the AD data set with several established gene lists associated with Alzheimer’s disease, obtained in different Alzheimer’s disease studies (Genotator [11], AlzGene [12], Soler et al. [13], Goni et al. [14]) and well known databases (KEGG [10], GeneCards (http://www.genecards.org)).

A standard over-representation analysis (ORA) then reveals whether the gene sets selected by the three methods are over- or under-represented in the established gene lists. Taking the approach of using the hypergeometric distribution as in [58] we estimate how likely the observations are due to chance. As reference set we use the set of all genes that are annotated on the Affymetrix array.

### Gene interactions

We examine *pairs* of genes that occur in combination in the same small gene set selected by the GA/SVM. We run the GA/SVM 3,000 times on the whole 1,000 gene data set to obtain 3,000 small sets of genes. Each of these small sets consists of 4 to 15 genes for AD and 2 to 8 genes for PLURI. The number of different gene pairs for a small set of *n* genes can be calculated as

(2) $$\frac{n\cdot (n-1)}{2}.$$

In the following, we define a joint occurrence as the co-occurrence of two genes *i* and *j* in the same small set of genes found by the GA/SVM. The actual number of such co-occurrences in the 3,000 examined small sets is denoted *k*_*i*,*j*_, and the number of occurrences of a single gene *i* is denoted *k*_*i*_.

Based on the single gene occurrences *k*_*i*_and *k*_*j*_, the expected frequency of a joint occurrence of genes *i* and *j*, assuming independence of the single occurrences, is defined as

(3) $$k_{i,j}^{\prime }=\frac{k_{i}k_{j}}{3000}\text{.}$$

To compute the statistical significance of the joint occurrences, we apply a *χ*^2^-test [59,60] with 1 degree of freedom and correct the resulting p-values using false discovery rate correction [46], using a cutoff of 0.05 for the estimated false discovery rate. As one requirement of the *χ*^2^-test, the expectation value $\left(k_{i,j}^{\prime }\right)$ has to be larger than 5 [61], so we also dismiss all joint occurrences that do not fit this criterion.

As a measurement for the importance of a joint occurrence we use the log-scaled ratio between the actual and the expected number of occurrences.

(4) $${\text{importance}\_\mathrm{jo}}_{i,j}=\log\left(\frac{k_{i,j}}{\overset{\prime }{\underset{i,j}{k}}}\right)$$

If $\left(k_{i,j}<k_{i,j}^{\prime }\right)$ we refer to the gene pair *i,j* as an under-represented gene pair, otherwise as an over-represented gene pair. So, the most over-represented gene pair is the gene pair with the largest value for *importance*_*jo*_*i*,*j*_ and the most under-represented gene pair is the pair with the smallest value.

To evaluate the obtained joint occurrences, we use three reference measurements defined by an SVM with Gaussian kernel. We classify the samples of the data sets with an SVM using the gene expression values of each gene *i* separately and define the resulting accuracy as *SVMacc*_*i*_; calculated by a 10-fold cross-validation on the whole set of samples. In the same way we determine *SVMacc*_*i*,*j*_using the gene expression values of both genes *i* and *j* as input. Our three reference values are then calculated as follows: 

1. SVM classification accuracy:

(5) $${\text{SVMacc}}_{i,j}$$

2. Mean gain of accuracy:

(6) $${\text{SVMgainMean}}_{i,j}={\text{SVMacc}}_{i,j}-\frac{{\text{SVMacc}}_{i}+{\text{SVMacc}}_{j}}{2}$$

3. Minimal gain of accuracy:

(7) $$\begin{array}{l} {\text{SVMgainMin}}_{i,j}={\text{SVMacc}}_{i,j}\\ \;-\text{max}\{{\text{SVMacc}}_{i},{\text{SVMacc}}_{j}\} \end{array}$$

We accumulate gene pairs by increasing and decreasing *importance*_*jo*_*i*,*j*_ and plot the three reference values to figure out whether there is a relationship between the classification accuracy of a gene pair (relative to the single genes) and the frequency of its choice by our GA/SVM.

## Competing interests

The authors declare that they have no competing interests.

## Authors’ contributions

L.S. carried out classification and feature selection and evaluated the results of the feature selection methods, performed enrichment analyses and wrote parts of the manuscript. M.L. contributed to the design of feature selection evaluation and improved the manuscript. R.S. improved the manuscript. D.R. carried out the calculation of mutual information, contributed to the design of cross-validation and gene set enrichment analysis and wrote parts of the manuscript. G.F. coordinated the study and wrote parts of the manuscript. All authors read and approved the final manuscript.

## Supplementary Material

Additional file 1 Blast search for LOC642711. Click here for file

Additional file 2 Comparison of the results obtained on the PLURI data set without the correct partitioning, the PLURI data set and the AD data set. Click here for file

Additional file 3 Partitioning of the AD data set. Click here for file

Additional file 4 Partitioning of the PLURI data set. Click here for file
