# Supplementary material for: Tissue-based Alzheimer gene expression markers–comparison of multiple machine learning approaches and investigation of redundancy in small biomarker sets
Source: BMC Bioinformatics. 2012 Oct 15;13:266. doi: 10.1186/1471-2105-13-266 (PMC3574043; doi:10.1186/1471-2105-13-266)
Supplement: Additional file 1 — Blast search for LOC642711. [file 1471-2105-13-266-S1.pdf]

BLAST ®

Basic Local Alignment Search Tool

NCBI/ BLAST/ blastn suite/ **Formatting Results - KZCNDJ5G01S**

Formatting options
Download

ref|XM_931285.1| (1585 letters)

Query ID gi|89038182|ref|XM_931285.1|
Description PREDICTED: Homo sapiens similar to junction-mediating and regulatory protein, transcript variant 5 (LOC642711), mRNA
Molecule type rna
Query Length 1585

Database Name Human G+T (2 databases)
Description
Program BLASTN 2.2.26+

Graphic Summary

Distribution of 58 Blast Hits on the Query Sequence

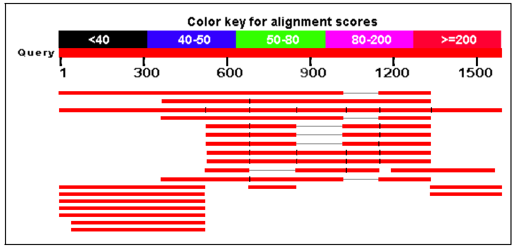

NCBI Blast:ref|XM_931285.1| (1585 letters) http://blast.ncbi.nlm.nih.gov/Blast.cgi#

Descriptions

Legend for links to other resources: U UniGene E GEO G Gene S Structure M Map Viewer PubChem BioAssay

Accession	Description	Max score	Total score	Query coverage	E value	Max ident	Links
**Transcripts**							
NR_036650.1	Homo sapiens WAS protein homolog associated with actin, golgi membranes and microtubules pseudogene (LOC100288615), non-coding RNA	1879	2225	75%	0.0	100%	G M
NR_003521.1	Homo sapiens WAS protein homolog associated with actin, golgi membranes and microtubules pseudogene 3 (WHAMMP3), non-coding RNA	1142	1614	60%	0.0	98%	E G M
**Genomic sequences[show first]**							
NT_010194.17	Homo sapiens chromosome 15 genomic contig, GRCh37.p5 Primary Assembly	968	4928	100%	0.0	100%	
**Transcripts[show first]**							
NM_001080435.1	Homo sapiens WAS protein homolog associated with actin, golgi membranes and microtubules (WHAMM), mRNA	944	1202	52%	0.0	93%	U E G M
**Genomic sequences[show first]**							
NT_078094.2	Homo sapiens chromosome 15 genomic contig, GRCh37.p5 Primary Assembly	329	1081	40%	1e-88	100%	
NW_001838191.2	Homo sapiens chromosome 15 genomic contig, alternate assembly HuRef SCAF_1103279188272, whole genome shotgun sequence	329	1081	40%	1e-88	100%	
NT_026446.14	Homo sapiens chromosome 15 genomic contig, GRCh37.p5 Primary Assembly	329	1092	40%	1e-88	100%	
NW_001838221.1	Homo sapiens chromosome 15 genomic contig, alternate assembly HuRef SCAF_1103279188173, whole genome shotgun sequence	303	1277	50%	8e-81	98%	
NT_077661.3	Homo sapiens chromosome 15 genomic contig, GRCh37.p5 Primary Assembly	303	1277	50%	8e-81	98%	
**Transcripts[show first]**							
XR_132589.1	PREDICTED: Homo sapiens u3 small nucleolar ribonucleoprotein protein MPP10-like (LOC100652840), miscRNA	676	676	23%	0.0	99%	G M
NR_026589.1	Homo sapiens WAS protein homolog associated with actin, golgi membranes and microtubules pseudogene 2 (WHAMMP2), non-coding RNA	599	1327	52%	9e-170	99%	E G M
**Genomic sequences[show first]**							
NW_001838205.1	Homo sapiens chromosome 15 genomic contig, alternate assembly HuRef SCAF_1103279183344, whole genome shotgun sequence	315	850	28%	4e-84	100%	G
NT_010194.17	Homo sapiens chromosome 15 genomic contig, GRCh37.p5 Primary Assembly	968	968	33%	0.0	100%	
NW_001841731.1	Homo sapiens unplaced genomic contig, alternate assembly HuRef DEGEN_1103279082069, whole genome shotgun sequence	968	968	33%	0.0	100%	G
NT_078094.2	Homo sapiens chromosome 15 genomic contig, GRCh37.p5 Primary Assembly	874	874	33%	0.0	97%	
NW_001838191.2	Homo sapiens chromosome 15 genomic contig, alternate assembly HuRef SCAF_1103279188272, whole genome shotgun sequence	874	874	33%	0.0	97%	
NT_026446.14	Homo sapiens chromosome 15 genomic contig, GRCh37.p5 Primary Assembly	863	863	33%	0.0	96%	
NW_001838221.1	Homo sapiens chromosome 15 genomic contig, alternate assembly HuRef SCAF_1103279188173, whole genome shotgun sequence	706	706	30%	0.0	93%	
NT_077661.3	Homo sapiens chromosome 15 genomic contig, GRCh37.p5 Primary Assembly	701	701	30%	0.0	93%	
NT_011362.10	Homo sapiens chromosome 20 genomic contig, GRCh37.p5 Primary Assembly	396	396	16%	1e-108	95%	
NW_001838666.1	Homo sapiens chromosome 20 genomic contig, alternate assembly HuRef SCAF_1103279188360, whole genome shotgun sequence	396	396	16%	1e-108	95%	
NW_001838205.1	Homo sapiens chromosome 15 genomic contig, alternate assembly HuRef SCAF_1103279183344, whole genome shotgun sequence	320	320	10%	8e-86	100%	G

Alignments

>ref|NR_036650.1| Homo sapiens WAS protein homolog associated with actin, golgi membranes and microtubules pseudogene (LOC100288615), non-coding RNA
Length=1567

GENE ID: 100288615 LOC100288615 | WAS protein homolog associated with actin, golgi membranes and microtubules pseudogene [Homo sapiens]
(10 or fewer PubMed links)

Sort alignments for this subject sequence by:
E value **Score** **Percent identity**
Query start position **Subject start position**

```
 Score = 1879 bits (1017),  Expect = 0.0
 Identities = 1017/1017 (100%), Gaps = 0/1017 (0%)
 Strand=Plus/Plus

Query  1     GTTGGTCAGGCTGGTGTCGAAGTCCTGACCTCAAGTGATCCACCCGCCTCGGCCTCCCAA  60
             ||||||||||||||||||||||||||||||||||||||||||||||||||||||||||||
Sbjct  7     GTTGGTCAGGCTGGTGTCGAAGTCCTGACCTCAAGTGATCCACCCGCCTCGGCCTCCCAA  66

Query  61    AGTGCTGGAATTACAGGCGTGAGCCACGGTGCCTGGCCAAAAGTCTCCATATTATTAAAC  120
             ||||||||||||||||||||||||||||||||||||||||||||||||||||||||||||
Sbjct  67    AGTGCTGGAATTACAGGCGTGAGCCACGGTGCCTGGCCAAAAGTCTCCATATTATTAAAC  126

Query  121   AATCTTCAGAAGCACAGTGCTGAATGACTACAGTAATAATATTCTGCCATGGATATATCA  180
             ||||||||||||||||||||||||||||||||||||||||||||||||||||||||||||
Sbjct  127   AATCTTCAGAAGCACAGTGCTGAATGACTACAGTAATAATATTCTGCCATGGATATATCA  186

Query  181   TAATTTACTTAACAATTCTTGTTTTATTGGGCATTTTTGATGGAGGATGATGACATTTTC  240
             ||||||||||||||||||||||||||||||||||||||||||||||||||||||||||||
Sbjct  187   TAATTTACTTAACAATTCTTGTTTTATTGGGCATTTTTGATGGAGGATGATGACATTTTC  246

Query  241   ATATTTAATCAATATTTTAAATTGATGTATTGAAAGTTGAGAACATGAAGGTTTCTTTTG  300
             ||||||||||||||||||||||||||||||||||||||||||||||||||||||||||||
Sbjct  247   ATATTTAATCAATATTTTAAATTGATGTATTGAAAGTTGAGAACATGAAGGTTTCTTTTG  306

Query  301   TTTAGTTTTGTTTGTTGGGTATGTATTACACTGTCTGACTTGAGCTTTATTCACATTTGC  360
             ||||||||||||||||||||||||||||||||||||||||||||||||||||||||||||
Sbjct  307   TTTAGTTTTGTTTGTTGGGTATGTATTACACTGTCTGACTTGAGCTTTATTCACATTTGC  366

Query  361   TCTCTAGGTTATCCAAGGACATGGAAAAGCCAACACCATGGTAGCATTAATGAAAGTTTA  420
             ||||||||||||||||||||||||||||||||||||||||||||||||||||||||||||
Sbjct  367   TCTCTAGGTTATCCAAGGACATGGAAAAGCCAACACCATGGTAGCATTAATGAAAGTTTA  426

Query  421   CCAAGAGGAAGATGAAGCCTACCAGGAATTAGTTACCATGGCAACCATGTTTTTCCAGTA  480
             ||||||||||||||||||||||||||||||||||||||||||||||||||||||||||||
Sbjct  427   CCAAGAGGAAGATGAAGCCTACCAGGAATTAGTTACCATGGCAACCATGTTTTTCCAGTA  486

Query  481   CTTACTGCAGCCATTTAGGGCTATGCGAGAAGTTGCAACTTTATAATTCCTTGGATGAGG  540
             ||||||||||||||||||||||||||||||||||||||||||||||||||||||||||||
Sbjct  487   CTTACTGCAGCCATTTAGGGCTATGCGAGAAGTTGCAACTTTATAATTCCTTGGATGAGG  546

Query  541   ATGACGTAGGTCCTACGTCTCCAGGATAGTTGCCCTGGAGAAAGAAGCTGAAGAATGGAC  600
             ||||||||||||||||||||||||||||||||||||||||||||||||||||||||||||
Sbjct  547   ATGACGTAGGTCCTACGTCTCCAGGATAGTTGCCCTGGAGAAAGAAGCTGAAGAATGGAC  606

Query  601   CAGACGGGCTGAAGAAGCTGTTGTCTCTATTCAAGATATCACAGTGAATTATTTTAATTA  660
             ||||||||||||||||||||||||||||||||||||||||||||||||||||||||||||
Sbjct  607   CAGACGGGCTGAAGAAGCTGTTGTCTCTATTCAAGATATCACAGTGAATTATTTTAATTA  666

Query  661   GACAGTAAAAGCATTAGCAGGAATGCAAAAAGAAATGGAACAGGATGTGAAGAGATTTGG  720
             ||||||||||||||||||||||||||||||||||||||||||||||||||||||||||||
Sbjct  667   GACAGTAAAAGCATTAGCAGGAATGCAAAAAGAAATGGAACAGGATGTGAAGAGATTTGG  726

Query  721   CCAGGCTGCCTGGGCCACAGCAATTCCCAGGTTGGAAAAACTTAAGCTAATGCTAGCTCA  780
             ||||||||||||||||||||||||||||||||||||||||||||||||||||||||||||
Sbjct  727   CCAGGCTGCCTGGGCCACAGCAATTCCCAGGTTGGAAAAACTTAAGCTAATGCTAGCTCA  786

Query  781   AGAGACTCTGCAACTCATGAGAGCGAAAGAATTGTATTTAAATCGCAAAAGAGCTGAAAT  840
             ||||||||||||||||||||||||||||||||||||||||||||||||||||||||||||
Sbjct  787   AGAGACTCTGCAACTCATGAGAGCGAAAGAATTGTATTTAAATCGCAAAAGAGCTGAAAT  846

Query  841   TCAGGGAAAGATGGAAGATCTTCCAGAACAAGAAAAAAATATAAATGTTGTAGATGAATT  900
             ||||||||||||||||||||||||||||||||||||||||||||||||||||||||||||
Sbjct  847   TCAGGGAAAGATGGAAGATCTTCCAGAACAAGAAAAAAATATAAATGTTGTAGATGAATT  906

Query  901   AGAAATGCAATTTTATGAAATTCAGTTAGAACTATATGAAGTTAAATTTGAGATATTAAA  960
             ||||||||||||||||||||||||||||||||||||||||||||||||||||||||||||
Sbjct  907   AGAAATGCAATTTTATGAAATTCAGTTAGAACTATATGAAGTTAAATTTGAGATATTAAA  966

Query  961   AAACGAAGAAATACTGCTTACTACACAGTTGGACTCTCTTAAAAGACTTATAAAAGA  1017
             |||||||||||||||||||||||||||||||||||||||||||||||||||||||||
Sbjct  967   AAACGAAGAAATACTGCTTACTACACAGTTGGACTCTCTTAAAAGACTTATAAAAGA  1023
```



```
 Score =  346 bits (187),  Expect = 1e-93
 Identities = 187/187 (100%), Gaps = 0/187 (0%)
 Strand=Plus/Plus

Query  1146  GAAAAACAGGATGAAGTTGTCTATTACGATCCATGTGAAAGTCCAGAGGAACTTAGTCAT  1205
             ||||||||||||||||||||||||||||||||||||||||||||||||||||||||||||
Sbjct  1022  GAAAAACAGGATGAAGTTGTCTATTACGATCCATGTGAAAGTCCAGAGGAACTTAGTCAT  1081

Query  1206  TGACTGTGGTGGGGCTGCAGGACGATAAGAATTCGGAAGTGAAAGAACTCAGAAGGCAGT  1265
             ||||||||||||||||||||||||||||||||||||||||||||||||||||||||||||
Sbjct  1082  TGACTGTGGTGGGGCTGCAGGACGATAAGAATTCGGAAGTGAAAGAACTCAGAAGGCAGT  1141

Query  1266  GCCAGCAGCTGGAGTCTGTTAAACGGGGCAGTCTGTGTCAAAAGAGCTTCTCTCCAGAGT  1325
             ||||||||||||||||||||||||||||||||||||||||||||||||||||||||||||
Sbjct  1142  GCCAGCAGCTGGAGTCTGTTAAACGGGGCAGTCTGTGTCAAAAGAGCTTCTCTCCAGAGT  1201

Query  1326  AGAAAGA  1332
             |||||||
Sbjct  1202  AGAAAGA  1208
```

>ref|NR_003521.1| Homo sapiens WAS protein homolog associated with actin, golgi membranes and microtubules pseudogene 3 (WHAMMP3), non-coding RNA
Length=3589

GENE ID: 339005 WHAMMP3 | WAS protein homolog associated with actin, golgi membranes and microtubules pseudogene 3 [Homo sapiens]
(10 or fewer PubMed links)

Sort alignments for this subject sequence by:
E value **Score** **Percent identity**
Query start position **Subject start position**

```
 Score = 1142 bits (618),  Expect = 0.0
 Identities = 643/655 (98%), Gaps = 2/655 (0%)
 Strand=Plus/Plus

Query  679   AGGAATGCAAAAAGAAATGGAACAGGATGTGAAGAGATTTGGCCAGGCTGCCTGGGCCAC  738
             ||||||||| ||||||||||||||||||||||||||||||||||||||||||||||||||
Sbjct  1062  AGGAATGCAGAAAGAAATGGAACAGGATGTGAAGAGATTTGGCCAGGCTGCCTGGGCCAC  1121

Query  739   AGCAATTCCCAGGTTGGAAAAACTTAAGCTAATGCTAGCTCAAGAGACTCTGCAACTCAT  798
             ||||||||||||||||||||||||||||||||||||||||||||||||||||||||||||
Sbjct  1122  AGCAATTCCCAGGTTGGAAAAACTTAAGCTAATGCTAGCTCAAGAGACTCTGCAACTCAT  1181

Query  799   GAGAGCGAAAGAATTGTATTTAAATCGCAAAAGAGCTGAAATTCAGGGAAAGATGGAAGA  858
             |||||||||||| || | |||||||| |||||||||||||||||||||||||||||||||
Sbjct  1182  GAGAGCGAAAGAGTTATGTTTAAATCACAAAAGAGCTGAAATTCAGGGAAAGATGGAAGA  1241

Query  859   TCTTCCAGAACAAGAAAAAAATATAAATGTTGTAGATGAATTAGAAATGCAATTTTATGA  918
             |||||||||||||||||||||||||||||||||||||||||||| ||| |||||||||||
Sbjct  1242  TCTTCCAGAACAAGAAAAAAATATAAATGTTGTAGATGAATTAGCAATACAATTTTATGA  1301

Query  919   AATTCAGTTAGAACTATATGAAGTTAAATTTGAGATATTAAAAACGAAGAAATACTGCT  978
             |||||||||||||||||||||||||||||||||||||||||||||| |||||||||||||
Sbjct  1302  AATTCAGTTAGAACTATATGAAGTTAAATTTGAGATATTAAAAAACAAAGAAATACTGCT  1361

Query  979   TACTACACAGTTGGACTCTCTTAAAAGACTTATAAAAGATGAAATTTAGAATCAAGTTCA  1038
             |||||||||||||||||||||| |||||||||||||||||||||||||||||||||||||
Sbjct  1362  TACTACACAGTTGGACTCTCTTGAAAGACTTATAAAAGATGAAATTTAGAATCAAGTTCA  1421

Query  1039  AAACAAAAAACTCTTTGGAATTTTGATTGTGATTTTGCTTAAAATTAGAGATTACTTTGG  1098
             ||||||||||||||||||||||||||||||||||||||||||||||||||||||||||||
Sbjct  1422  AAACAAAAAACTCTTTGGAATTTTGATTGTGATTTTGCTTAAAATTAGAGATTACTTTGG  1481

Query  1099  GGAGAATAGTGGTCTTTGCAATTTTGAATCTTCCTACCCAAGAACATGAAAAACAGGATG  1158
             ||||||||||||||||||||||||||||||||||||||||||||||||||||||||||||
Sbjct  1482  GGAGAATAGTGGTCTTTGCAATTTTGAATCTTCCTACCCAAGAACATGAAAAACAGGATG  1541

Query  1159  AAGTTGTCTATTACGATCCATGTGAAAGTCCAGAGGAACTTAGTCATTGACTGTGGTGGG  1218
             ||||||||||||||||||||||||||||||||||||||||||||||||||||||||||||
Sbjct  1542  AAGTTGTCTATTACGATCCATGTGAAAGTCCAGAGGAACTTAGTCATTGACTGTGGTGGG  1601

Query  1219  GCTGCAGGACGATAAGAATTCGGAAGTGAAAGAACTCAGAAGGCAGTGCCAGCAGCTGGA  1278
             ||||||||||||||||||||||||||||||||||||||||||||||||||||||||||||
Sbjct  1602  GCTGCAGGACGATAAGAATTCGGAAGTGAAAGAACTCAGAAGGCAGTGCCAGCAGCTGGA  1661

Query  1279  GTCTGTTAAACGGGGCAG--TCTGTGTCAAAAGAGCTTCTCTCCAGAGTAGAAAG  1331
             |||| |||||||||||||  |||||||||||||||||||||||||||||||||||
Sbjct  1662  GTCTATTAAACGGGGCAGGATCTGTGTCAAAAGAGCTTCTCTCCAGAGTAGAAAG  1716


 Score =  472 bits (255),  Expect = 2e-131
 Identities = 296/315 (94%), Gaps = 5/315 (2%)
 Strand=Plus/Plus

Query  368   GTTATCCAAGGACATGGAAAAGCCAACACCATGGTAGCATTAATGAAAGTTTACCAAGAG  427
             ||||| ||||||||  ||||||||||||||||||||||||||||||||||||||||||||
Sbjct  597   GTTATTCAAGGACACCGAAAAGCCAACACCATGGTAGCATTAATGAAAGTTTACCAAGAG  656

Query  428   GAAGATGAAGCCTACCAGGAATTAGTTACCATGGCAACCATGTTTTTCCAGTACTTACTG  487
             |||||||||||||||||||||||||||||| ||||||||| ||| |||||||||||||||
Sbjct  657   GAAGATGAAGCCTACCAGGAATTAGTTACCGTGGCAACCACGTTCTTCCAGTACTTACTG  716

Query  488   CAGCCATTTAGGGCTATGCGAGAAGTTGCAACTTTATAATTCCTTGGATGAGGATGACGT  547
             ||||||||||||||||||||||||||||||||||||||| |||||||||||||||||| |
Sbjct  717   CAGCCATTTAGGGCTATGCGAGAAGTTGCAACTTTATAAGTCCTTGGATGAGGATGACCT  776

Query  548   AGGTCCTACGTCTCCAGGATAGTTGCCCTGGAGAAAGAAGCTGAAGAATGGACCAGACGG  607
             |||||||| |     ||| |||||||||||||||||||||||||||||||||||||||||
Sbjct  777   AGGTCCTA-GA----AGGGTAGTTGCCCTGGAGAAAGAAGCTGAAGAATGGACCAGACGG  831
```

```
Query  608   GCTGAAGAAGCTGTTGTCTCTATTCAAGATATCACAGTGAATTATTTAATTAGACAGTA  667
             |||| ||||||||| |||||||||||||||||||||||||||||||||||  ||||||||
Sbjct  832   GCTGGAGAAGCTGTCGTCTCTATTCAAGATATCACAGTGAATTATTTAAGGAGACAGTA  891

Query  668   AAAGCATTAGCAGGA  682
             |||||||||||||||
Sbjct  892   AAAGCATTAGCAGGA  906
```

>**ref|NT_010194.17|** D Homo sapiens chromosome 15 genomic contig, GRCh37.p5 Primary Assembly
Length=53620202

Sort alignments for this subject sequence by:
E value **Score** **Percent identity**
Query start position **Subject start position**

Features flanking this part of subject sequence:
78470 bp at 5' side: golgin subfamily A member 8-like protein 2-like
59819 bp at 3' side: golgin subfamily A member 8-like isoform 1

Score = 968 bits (524), Expect = 0.0
Identities = 524/524 (100%), Gaps = 0/524 (0%)
Strand=Plus/Minus

```
Query  1        GTTGGTCAGGCTGGTGTCGAAGTCCTGACCTCAAGTGATCCACCCGCCTCGGCCTCCCAA  60
                ||||||||||||||||||||||||||||||||||||||||||||||||||||||||||||
Sbjct  3616493  GTTGGTCAGGCTGGTGTCGAAGTCCTGACCTCAAGTGATCCACCCGCCTCGGCCTCCCAA  3616434

Query  61       AGTGCTGGAATTACAGGCGTGAGCCACGGTGCCTGGCCAAAAGTCTCCATATTATTAAAC  120
                ||||||||||||||||||||||||||||||||||||||||||||||||||||||||||||
Sbjct  3616433  AGTGCTGGAATTACAGGCGTGAGCCACGGTGCCTGGCCAAAAGTCTCCATATTATTAAAC  3616374

Query  121      AATCTTCAGAAGCACAGTGCTGAATGACTACAGTAATAATATTCTGCCATGGATATATCA  180
                ||||||||||||||||||||||||||||||||||||||||||||||||||||||||||||
Sbjct  3616373  AATCTTCAGAAGCACAGTGCTGAATGACTACAGTAATAATATTCTGCCATGGATATATCA  3616314

Query  181      TAATTTACTTAACAATTCTTGTTTTATTGGGCATTTTTGATGGAGGATGATGACATTTTC  240
                ||||||||||||||||||||||||||||||||||||||||||||||||||||||||||||
Sbjct  3616313  TAATTTACTTAACAATTCTTGTTTTATTGGGCATTTTTGATGGAGGATGATGACATTTTC  3616254

Query  241      ATATTTAATCAATATTTTAAATTGATGTATTGAAAGTTGAGAACATGAAGGTTTCTTTTG  300
                ||||||||||||||||||||||||||||||||||||||||||||||||||||||||||||
Sbjct  3616253  ATATTTAATCAATATTTTAAATTGATGTATTGAAAGTTGAGAACATGAAGGTTTCTTTTG  3616194

Query  301      TTTAGTTTTGTTTGTTGGGTATGTATTACACTGTCTGACTTGAGCTTTATTCACATTTGC  360
                ||||||||||||||||||||||||||||||||||||||||||||||||||||||||||||
Sbjct  3616193  TTTAGTTTTGTTTGTTGGGTATGTATTACACTGTCTGACTTGAGCTTTATTCACATTTGC  3616134

Query  361      TCTCTAGGTTATCCAAGGACATGGAAAAGCCAACACCATGGTAGCATTAATGAAAGTTTA  420
                ||||||||||||||||||||||||||||||||||||||||||||||||||||||||||||
Sbjct  3616133  TCTCTAGGTTATCCAAGGACATGGAAAAGCCAACACCATGGTAGCATTAATGAAAGTTTA  3616074

Query  421      CCAAGAGGAAGATGAAGCCTACCAGGAATTAGTTACCATGGCAACCATGTTTTTCCAGTA  480
                ||||||||||||||||||||||||||||||||||||||||||||||||||||||||||||
Sbjct  3616073  CCAAGAGGAAGATGAAGCCTACCAGGAATTAGTTACCATGGCAACCATGTTTTTCCAGTA  3616014

Query  481      CTTACTGCAGCCATTTAGGGCTATGCGAGAAGTTGCAACTTTAT  524
                ||||||||||||||||||||||||||||||||||||||||||||
Sbjct  3616013  CTTACTGCAGCCATTTAGGGCTATGCGAGAAGTTGCAACTTTAT  3615970
```

Features flanking this part of subject sequence:
59126 bp at 5' side: golgin subfamily A member 8-like protein 2-like
79431 bp at 3' side: golgin subfamily A member 8-like isoform 1

Score = 473 bits (256), Expect = 5e-132
Identities = 256/256 (100%), Gaps = 0/256 (0%)
Strand=Plus/Minus

```
Query  1330     AGATTTATTTTCTTTTTGAATATATTTTCTACACCATGGTTTCTCAACCAGAGGTGATAC  1389
                ||||||||||||||||||||||||||||||||||||||||||||||||||||||||||||
Sbjct  3596881  AGATTTATTTTCTTTTTGAATATATTTTCTACACCATGGTTTCTCAACCAGAGGTGATAC  3596822

Query  1390     AGCACTGTCTCCAAATGTGGGGCTGTTGTGTATTGTCACGGGGAGGAGAAATGCTACTGG  1449
                ||||||||||||||||||||||||||||||||||||||||||||||||||||||||||||
Sbjct  3596821  AGCACTGTCTCCAAATGTGGGGCTGTTGTGTATTGTCACGGGGAGGAGAAATGCTACTGG  3596762

Query  1450     CATTTAGTGGATGATGGTCAAGGACACTAAGCAACCTGCAATGTGTGGATCAATCTTACA  1509
                ||||||||||||||||||||||||||||||||||||||||||||||||||||||||||||
Sbjct  3596761  CATTTAGTGGATGATGGTCAAGGACACTAAGCAACCTGCAATGTGTGGATCAATCTTACA  3596702

Query  1510     CAACGAAGGACTGTTCCACCCCAAATGCCAATGGTACCCTGTTGAGAAAGGTTAGTCTAT  1569
                ||||||||||||||||||||||||||||||||||||||||||||||||||||||||||||
Sbjct  3596701  CAACGAAGGACTGTTCCACCCCAAATGCCAATGGTACCCTGTTGAGAAAGGTTAGTCTAT  3596642

Query  1570     GTGGACAGTGAATTAT  1585
                ||||||||||||||||
Sbjct  3596641  GTGGACAGTGAATTAT  3596626
```

Features flanking this part of subject sequence:
79425 bp at 5' side: golgin subfamily A member 8-like protein 2-like
110284 bp at 3' side: golgin subfamily A member 8H isoform 1

Score = 468 bits (253), Expect = 3e-130
Identities = 255/256 (99%), Gaps = 0/256 (0%)
Strand=Plus/Plus

```
Query  1330     AGATTTATTTTCTTTTTGAATATATTTTCTACACCATGGTTTCTCAACCAGAGGTGATAC  1389
                ||||||||||||||||||||||||||||||||||||||||||||||||||||||||||||
Sbjct  1576347  AGATTTATTTTCTTTTTGAATATATTTTCTACACCATGGTTTCTCAACCAGAGGTGATAC  1576406
```



```
Query  1390     AGCACTGTCTCCAAATGTGGGGCTGTTGTGTATTGTCACGGGGAGGAGAAATGCTACTGG  1449
                |||||||||||||||||||| |||||||||||||||||||||||||||||||||||||||
Sbjct  1576407  AGCACTGTCTCCAAATGTGGAGCTGTTGTGTATTGTCACGGGGAGGAGAAATGCTACTGG  1576466

Query  1450     CATTTAGTGGATGATGGTCAAGGACACTAAGCAACCTGCAATGTGTGGATCAATCTTACA  1509
                ||||||||||||||||||||||||||||||||||||||||||||||||||||||||||||
Sbjct  1576467  CATTTAGTGGATGATGGTCAAGGACACTAAGCAACCTGCAATGTGTGGATCAATCTTACA  1576526

Query  1510     CAACGAAGGACTGTTCCACCCCAAATGCCAATGGTACCCTGTTGAGAAAGGTTAGTCTAT  1569
                ||||||||||||||||||||||||||||||||||||||||||||||||||||||||||||
Sbjct  1576527  CAACGAAGGACTGTTCCACCCCAAATGCCAATGGTACCCTGTTGAGAAAGGTTAGTCTAT  1576586

Query  1570     GTGGACAGTGAATTAT  1585
                ||||||||||||||||
Sbjct  1576587  GTGGACAGTGAATTAT  1576602
```

Features flanking this part of subject sequence:
68287 bp at 5' side: golgin subfamily A member 8-like protein 2-like
70340 bp at 3' side: golgin subfamily A member 8-like isoform 1

Score = 344 bits (186), Expect = 4e-93
Identities = 186/186 (100%), Gaps = 0/186 (0%)
Strand=Plus/Minus

```
Query  1146     GAAAAACAGGATGAAGTTGTCTATTACGATCCATGTGAAAGTCCAGAGGAACTTAGTCAT  1205
                ||||||||||||||||||||||||||||||||||||||||||||||||||||||||||||
Sbjct  3605972  GAAAAACAGGATGAAGTTGTCTATTACGATCCATGTGAAAGTCCAGAGGAACTTAGTCAT  3605913

Query  1206     TGACTGTGGTGGGGCTGCAGGACGATAAGAATTCGGAAGTGAAAGAACTCAGAAGGCAGT  1265
                ||||||||||||||||||||||||||||||||||||||||||||||||||||||||||||
Sbjct  3605912  TGACTGTGGTGGGGCTGCAGGACGATAAGAATTCGGAAGTGAAAGAACTCAGAAGGCAGT  3605853

Query  1266     GCCAGCAGCTGGAGTCTGTTAAACGGGGCAGTCTGTGTCAAAAGAGCTTCTCTCCAGAGT  1325
                ||||||||||||||||||||||||||||||||||||||||||||||||||||||||||||
Sbjct  3605852  GCCAGCAGCTGGAGTCTGTTAAACGGGGCAGTCTGTGTCAAAAGAGCTTCTCTCCAGAGT  3605793

Query  1326     AGAAAG  1331
                ||||||
Sbjct  3605792  AGAAAG  3605787
```

Features flanking this part of subject sequence:
70339 bp at 5' side: golgin subfamily A member 8-like protein 2-like
119440 bp at 3' side: golgin subfamily A member 8H isoform 1

Score = 339 bits (183), Expect = 2e-91
Identities = 185/186 (99%), Gaps = 0/186 (0%)
Strand=Plus/Plus

```
Query  1146     GAAAAACAGGATGAAGTTGTCTATTACGATCCATGTGAAAGTCCAGAGGAACTTAGTCAT  1205
                ||||||||||||||||||||||||||||||||||||||||||||||||||||||||||||
Sbjct  1567261  GAAAAACAGGATGAAGTTGTCTATTACGATCCATGTGAAAGTCCAGAGGAACTTAGTCAT  1567320

Query  1206     TGACTGTGGTGGGGCTGCAGGACGATAAGAATTCGGAAGTGAAAGAACTCAGAAGGCAGT  1265
                ||||||||||||||||||||||||||||||||||||||||||||||||||||||||||||
Sbjct  1567321  TGACTGTGGTGGGGCTGCAGGACGATAAGAATTCGGAAGTGAAAGAACTCAGAAGGCAGT  1567380

Query  1266     GCCAGCAGCTGGAGTCTGTTAAACGGGGCAGTCTGTGTCAAAAGAGCTTCTCTCCAGAGT  1325
                |||||||||||||||||||||||||| |||||||||||||||||||||||||||||||||
Sbjct  1567381  GCCAGCAGCTGGAGTCTGTTAAACGGAGCAGTCTGTGTCAAAAGAGCTTCTCTCCAGAGT  1567440

Query  1326     AGAAAG  1331
                ||||||
Sbjct  1567441  AGAAAG  1567446
```

Features flanking this part of subject sequence:
73790 bp at 5' side: golgin subfamily A member 8-like protein 2-like
64850 bp at 3' side: golgin subfamily A member 8-like isoform 1

Score = 320 bits (173), Expect = 8e-86
Identities = 173/173 (100%), Gaps = 0/173 (0%)
Strand=Plus/Minus

```
Query  678      CAGGAATGCAAAAAGAAATGGAACAGGATGTGAAGAGATTTGGCCAGGCTGCCTGGGCCA  737
                ||||||||||||||||||||||||||||||||||||||||||||||||||||||||||||
Sbjct  3611462  CAGGAATGCAAAAAGAAATGGAACAGGATGTGAAGAGATTTGGCCAGGCTGCCTGGGCCA  3611403

Query  738      CAGCAATTCCCAGGTTGGAAAAACTTAAGCTAATGCTAGCTCAAGAGACTCTGCAACTCA  797
                ||||||||||||||||||||||||||||||||||||||||||||||||||||||||||||
Sbjct  3611402  CAGCAATTCCCAGGTTGGAAAAACTTAAGCTAATGCTAGCTCAAGAGACTCTGCAACTCA  3611343

Query  798      TGAGAGCGAAAGAATTGTATTTAAATCGCAAAAGAGCTGAAATTCAGGGAAAG  850
                |||||||||||||||||||||||||||||||||||||||||||||||||||||
Sbjct  3611342  TGAGAGCGAAAGAATTGTATTTAAATCGCAAAAGAGCTGAAATTCAGGGAAAG  3611290
```

Features flanking this part of subject sequence:
64859 bp at 5' side: golgin subfamily A member 8-like protein 2-like
124933 bp at 3' side: golgin subfamily A member 8H isoform 1

Score = 315 bits (170), Expect = 4e-84
Identities = 172/173 (99%), Gaps = 0/173 (0%)
Strand=Plus/Plus

```
Query  678      CAGGAATGCAAAAAGAAATGGAACAGGATGTGAAGAGATTTGGCCAGGCTGCCTGGGCCA  737
                |||||||||| |||||||||||||||||||||||||||||||||||||||||||||||||
Sbjct  1561781  CAGGAATGCAGAAAGAAATGGAACAGGATGTGAAGAGATTTGGCCAGGCTGCCTGGGCCA  1561840

Query  738      CAGCAATTCCCAGGTTGGAAAAACTTAAGCTAATGCTAGCTCAAGAGACTCTGCAACTCA  797
```

```
                      ||||||||||||||||||||||||||||||||||||||||||||||||||||||||||||
Sbjct  1561841  CAGCAATTCCCAGGTTGGAAAAACTTAAGCTAATGCTAGCTCAAGAGACTCTGCAACTCA  1561900

Query  798      TGAGAGCGAAAGAATTGTATTTAAATCGCAAAAGAGCTGAAATTCAGGGAAAG  850
                |||||||||||||||||||||||||||||||||||||||||||||||||||||
Sbjct  1561901  TGAGAGCGAAAGAATTGTATTTAAATCGCAAAAGAGCTGAAATTCAGGGAAAG  1561953
```

Features flanking this part of subject sequence:
72061 bp at 5' side: golgin subfamily A member 8-like protein 2-like
66573 bp at 3' side: golgin subfamily A member 8-like isoform 1

Score = 315 bits (170), Expect = 4e-84
Identities = 177/180 (98%), Gaps = 2/180 (1%)
Strand=Plus/Minus

```
Query  848      AAGATGGAAGATCTTCCAGAACAAGAAAAAAATATAAATGTTGTAGATGAATTAGAAATG  907
                ||||||||||||||||||||||||||||||||||||||||||||||||||||||||||||
Sbjct  3609739  AAGATGGAAGATCTTCCAGAACAAGAAAAAAATATAAATGTTGTAGATGAATTAGAAATG  3609680

Query  908      CAATTTTATGAAATTCAGTTAGAACTATATGAAGTTAAATTTGAGATATTAAAAAACGAA  967
                ||||||||||||||||||||||||||||||||||||||||||||||||||||||||||||
Sbjct  3609679  CAATTTTATGAAATTCAGTTAGAACTATATGAAGTTAAATTTGAGATATTAAAAAACGAA  3609620

Query  968      GAAATACTGCTTACTACACAGTTGGACTCTCTTAAAAGACTTATAAAAGATGAAA-TTTA  1026
                ||||||||||||||||||||||||||||||||||||||||||||||||| | ||| ||||
Sbjct  3609619  GAAATACTGCTTACTACACAGTTGGACTCTCTTAAAAGACTTATAAAAGGT-AAAGTTTA  3609561
```

Features flanking this part of subject sequence:
66577 bp at 5' side: golgin subfamily A member 8-like protein 2-like
123220 bp at 3' side: golgin subfamily A member 8H isoform 1

Score = 311 bits (168), Expect = 5e-83
Identities = 168/168 (100%), Gaps = 0/168 (0%)
Strand=Plus/Plus

```
Query  848      AAGATGGAAGATCTTCCAGAACAAGAAAAAAATATAAATGTTGTAGATGAATTAGAAATG  907
                ||||||||||||||||||||||||||||||||||||||||||||||||||||||||||||
Sbjct  1563499  AAGATGGAAGATCTTCCAGAACAAGAAAAAAATATAAATGTTGTAGATGAATTAGAAATG  1563558

Query  908      CAATTTTATGAAATTCAGTTAGAACTATATGAAGTTAAATTTGAGATATTAAAAAACGAA  967
                ||||||||||||||||||||||||||||||||||||||||||||||||||||||||||||
Sbjct  1563559  CAATTTTATGAAATTCAGTTAGAACTATATGAAGTTAAATTTGAGATATTAAAAAACGAA  1563618

Query  968      GAAATACTGCTTACTACACAGTTGGACTCTCTTAAAAGACTTATAAAA  1015
                ||||||||||||||||||||||||||||||||||||||||||||||||
Sbjct  1563619  GAAATACTGCTTACTACACAGTTGGACTCTCTTAAAAGACTTATAAAA  1563666
```

Features flanking this part of subject sequence:
63625 bp at 5' side: golgin subfamily A member 8-like protein 2-like
126183 bp at 3' side: golgin subfamily A member 8H isoform 1

Score = 291 bits (157), Expect = 6e-77
Identities = 157/157 (100%), Gaps = 0/157 (0%)
Strand=Plus/Plus

```
Query  525      AATTCCTTGGATGAGGATGACGTAGGTCCTACGTCTCCAGGATAGTTGCCCTGGAGAAAG  584
                ||||||||||||||||||||||||||||||||||||||||||||||||||||||||||||
Sbjct  1560547  AATTCCTTGGATGAGGATGACGTAGGTCCTACGTCTCCAGGATAGTTGCCCTGGAGAAAG  1560606

Query  585      AAGCTGAAGAATGGACCAGACGGGCTGAAGAAGCTGTTGTCTCTATTCAAGATATCACAG  644
                ||||||||||||||||||||||||||||||||||||||||||||||||||||||||||||
Sbjct  1560607  AAGCTGAAGAATGGACCAGACGGGCTGAAGAAGCTGTTGTCTCTATTCAAGATATCACAG  1560666

Query  645      TGAATTATTTTAATTAGACAGTAAAAGCATTAGCAGG  681
                |||||||||||||||||||||||||||||||||||||
Sbjct  1560667  TGAATTATTTTAATTAGACAGTAAAAGCATTAGCAGG  1560703
```

Features flanking this part of subject sequence:
75040 bp at 5' side: golgin subfamily A member 8-like protein 2-like
63616 bp at 3' side: golgin subfamily A member 8-like isoform 1

Score = 291 bits (157), Expect = 6e-77
Identities = 157/157 (100%), Gaps = 0/157 (0%)
Strand=Plus/Minus

```
Query  525      AATTCCTTGGATGAGGATGACGTAGGTCCTACGTCTCCAGGATAGTTGCCCTGGAGAAAG  584
                ||||||||||||||||||||||||||||||||||||||||||||||||||||||||||||
Sbjct  3612696  AATTCCTTGGATGAGGATGACGTAGGTCCTACGTCTCCAGGATAGTTGCCCTGGAGAAAG  3612637

Query  585      AAGCTGAAGAATGGACCAGACGGGCTGAAGAAGCTGTTGTCTCTATTCAAGATATCACAG  644
                ||||||||||||||||||||||||||||||||||||||||||||||||||||||||||||
Sbjct  3612636  AAGCTGAAGAATGGACCAGACGGGCTGAAGAAGCTGTTGTCTCTATTCAAGATATCACAG  3612577

Query  645      TGAATTATTTTAATTAGACAGTAAAAGCATTAGCAGG  681
                |||||||||||||||||||||||||||||||||||||
Sbjct  3612576  TGAATTATTTTAATTAGACAGTAAAAGCATTAGCAGG  3612540
```

Features flanking this part of subject sequence:
68955 bp at 5' side: golgin subfamily A member 8-like protein 2-like
120878 bp at 3' side: golgin subfamily A member 8H isoform 1

Score = 244 bits (132), Expect = 5e-63
Identities = 132/132 (100%), Gaps = 0/132 (0%)
Strand=Plus/Plus

```
Query  1015     AGATGAAATTTAGAATCAAGTTCAAAACAAAAAACTCTTTGGAATTTTGATTGTGATTTT  1074
```



```
                      ||||||||||||||||||||||||||||||||||||||||||||||||||||||||||||
Sbjct  1565877  AGATGAAATTTAGAATCAAGTTCAAAACAAAAAACTCTTTGGAATTTTGATTGTGATTTT  1565936

Query  1075     GCTTAAAATTAGAGATTACTTTGGGGAGAATAGTGGTCTTTGCAATTTTGAATCTTCCTA  1134
                ||||||||||||||||||||||||||||||||||||||||||||||||||||||||||||
Sbjct  1565937  GCTTAAAATTAGAGATTACTTTGGGGAGAATAGTGGTCTTTGCAATTTTGAATCTTCCTA  1565996

Query  1135     CCCAAGAACATG  1146
                ||||||||||||
Sbjct  1565997  CCCAAGAACATG  1566008
```

Features flanking this part of subject sequence:
69729 bp at 5' side: golgin subfamily A member 8-like protein 2-like
68952 bp at 3' side: golgin subfamily A member 8-like isoform 1

Score = 244 bits (132), Expect = 5e-63
Identities = 132/132 (100%), Gaps = 0/132 (0%)
Strand=Plus/Minus

```
Query  1015     AGATGAAATTTAGAATCAAGTTCAAAACAAAAAACTCTTTGGAATTTTGATTGTGATTTT  1074
                ||||||||||||||||||||||||||||||||||||||||||||||||||||||||||||
Sbjct  3607360  AGATGAAATTTAGAATCAAGTTCAAAACAAAAAACTCTTTGGAATTTTGATTGTGATTTT  3607301

Query  1075     GCTTAAAATTAGAGATTACTTTGGGGAGAATAGTGGTCTTTGCAATTTTGAATCTTCCTA  1134
                ||||||||||||||||||||||||||||||||||||||||||||||||||||||||||||
Sbjct  3607300  GCTTAAAATTAGAGATTACTTTGGGGAGAATAGTGGTCTTTGCAATTTTGAATCTTCCTA  3607241

Query  1135     CCCAAGAACATG  1146
                ||||||||||||
Sbjct  3607240  CCCAAGAACATG  3607229
```

>**ref|NM_001080435.1|** UEGM Homo sapiens WAS protein homolog associated with actin, golgi membranes and microtubules (WHAMM), mRNA
Length=4261

GENE ID: 123720 WHAMM | WAS protein homolog associated with actin, golgi membranes and microtubules [Homo sapiens] (10 or fewer PubMed links)

Sort alignments for this subject sequence by:
E value **Score** **Percent identity**
Query start position **Subject start position**

Score = 944 bits (511), Expect = 0.0
Identities = 620/669 (93%), Gaps = 22/669 (3%)
Strand=Plus/Plus

```
Query  366   AGGTTATCCAAGGACATGGAAAAGCCAACACCATGGTAGCATTAATGAAAGTTTACCAAG  425
             ||||||| |||||||| |||||||||||||||||||||||||||||||| ||||||||||
Sbjct  1114  AGGTTATTCAAGGACACGGAAAAGCCAACACCATGGTAGCATTAATGAACGTTTACCAAG  1173

Query  426   AGGAAGATGAAGCCTACCAGGAATTAGTTACCATGGCAACCATGTTTTTCCAGTACTTAC  485
             ||||||||||||| ||||||||||| |||||| ||||||||||||| |||||||||||| 
Sbjct  1174  AGGAAGATGAAGCATACCAGGAATTGGTTACCGTGGCAACCATGTTCTTCCAGTACTTAT  1233

Query  486   TGCAGCCATTTAGGGCTATGCGAGAAGTTGCAAC--T-T-T-A---T-A-A---T----T  528
             ||||||||||||||||||||||||||||||||||  | | | |   | | |   |    |
Sbjct  1234  TGCAGCCATTTAGGGCTATGCGAGAAGTTGCAACTTTATGTAAGCTTGATATTTTGAAGT  1293

Query  529   CCTTGGATGAGGATGACGTAGGTCCTACGTCTCCAGGATAGTTGCCCTGGAGAAAGAAGC  588
             | ||||||||||||||| ||||||||| |     ||| ||||||||||||||||||||||
Sbjct  1294  CTTTGGATGAGGATGACCTAGGTCCTA-G----AAGGGTAGTTGCCCTGGAGAAAGAAGC  1348

Query  589   TGAAGAATGGACCAGACGGGCTGAAGAAGCTGTTGTCTCTATTCAAGATATCACAGTGAA  648
             |||||||||||||||||||||||||||||||| |||||||||||| ||||||||||||||
Sbjct  1349  TGAAGAATGGACCAGACGGGCTGAAGAAGCTGTCGTCTCTATTCAGGATATCACAGTGAA  1408

Query  649   TTATTTTAATTAGACAGTAAAAGCATTAGCAGGAATGCAAAAAGAAATGGAACAGGATGT  708
             |||||||||  |||||||||||||||||||||||||||| ||||||||||||||||||| 
Sbjct  1409  TTATTTTAAGGAGACAGTAAAAGCATTAGCAGGAATGCAGAAAGAAATGGAACAGGATGC  1468

Query  709   GAAGAGATTTGGCCAGGCTGCCTGGGCCACAGCAATTCCCAGGTTGGAAAAACTTAAGCT  768
             |||||||||||| |||||||||||||||||||||||||||||||||||||||||| ||||
Sbjct  1469  GAAGAGATTTGGTCAGGCTGCCTGGGCCACAGCAATTCCCAGGTTGGAAAAACTTCAGCT  1528

Query  769   AATGCTAGCTCAAGAGACTCTGCAACTCATGAGAGCGAAAGAATTGTATTTAAATCGCAA  828
             ||||||||||| |||||||||||||||||||||||||||||| |||| |||||||| |||
Sbjct  1529  AATGCTAGCTCGAGAGACTCTGCAACTCATGAGAGCGAAAGAGTTGTGTTTAAATCACAA  1588

Query  829   AAGAGCTGAAATTCAGGGAAAGATGGAAGATCTTCCAGAACAAGAAAAAAATATAAATGT  888
             ||||||||||||||||||||||||||||||||||||||||||||||||||||| ||||||
Sbjct  1589  AAGAGCTGAAATTCAGGGAAAGATGGAAGATCTTCCAGAACAAGAAAAAAATACAAATGT  1648

Query  889   TGTAGATGAATTAGAAATGCAATTTTATGAAATTCAGTTAGAACTATATGAAGTTAAATT  948
             |||||||||||||||||| ||||||||||||||||| |||||||||||||||||||||||
Sbjct  1649  TGTAGATGAATTAGAAATACAATTTTATGAAATTCAATTAGAACTATATGAAGTTAAATT  1708

Query  949   TGAGATATTAAAAAACGAAGAAATACTGCTTACTACACAGTTGGACTCTCTTAAAAGACT  1008
             ||||||||||||||||||||||||||||||||||||||||||||||||||||||||||||
Sbjct  1709  TGAGATATTAAAAAACGAAGAAATACTGCTTACTACACAGTTGGACTCTCTTAAAAGACT  1768

Query  1009  TATAAAAGA  1017
             |||||||||
Sbjct  1769  TATAAAAGA  1777
```

Score = 257 bits (139), Expect = 6e-67
Identities = 176/192 (92%), Gaps = 9/192 (5%)
Strand=Plus/Plus

```
Query  1146  GAAAAACAGGATGAAGTTGTCTATTACGATCCATGTGAAAGTCCAGAGGAACTT--AGTC  1203
```

```
                ||||||||| ||||||||||||||||||||||||||||||| |||||||||||||  ||||
Sbjct  1776  GAAAAACAAGATGAAGTTGTCTATTACGATCCATGTGAAAATCCAGAGGAACTTAAAGTC  1835

Query  1204  ATTGAC--TGTGGTGGGGCTGCAGGACGATAAGAATTCGGAAGTGAAAGAACTCAGAAGG  1261
             ||||||  |||||||||||||||||| |||||||||| ||||||||||||||||||||||
Sbjct  1836  ATTGACTGTGTGGTGGGGCTGCAGGATGATAAGAATTTGGAAGTGAAAGAACTCAGAAGG  1895

Query  1262  CAGTGCCAGCAGCTGGAGTCTGTTAAACGGGGCAG--TCTGTGTCAAAAGAGCTTCTCTC  1319
             |||||||||||||||||||||   |||||||||||  |||||| ||||||||| ||||||
Sbjct  1896  CAGTGCCAGCAGCTGGAGTCT---AAACGGGGCAGGATCTGTGCCAAAAGAGCCTCTCTC  1952

Query  1320  CAGAGTAGAAAG  1331
             | ||||||||||
Sbjct  1953  CGGAGTAGAAAG  1964
```

>**ref|NT_078094.2|** D Homo sapiens chromosome 15 genomic contig, GRCh37.p5 Primary Assembly
Length=868660

Sort alignments for this subject sequence by:
E value **Score** **Percent identity**
Query start position **Subject start position**

Features flanking this part of subject sequence:
132441 bp at 5' side: magnesium transporter NIPA1 isoform 2
211371 bp at 3' side: putative golgin subfamily A member 6-like protein 6-like ...

Score = 329 bits (178), Expect = 1e-88
Identities = 185/188 (98%), Gaps = 2/188 (1%)
Strand=Plus/Minus

```
Query  1146    GAAAAACAGGATGAAGTTGTCTATTACGATCCATGTGAAAGTCCAGAGGAACTTAGTCAT  1205
               ||||||||||||||||||||||||||||||||||||||||||||||||||||||||||||
Sbjct  548707  GAAAAACAGGATGAAGTTGTCTATTACGATCCATGTGAAAGTCCAGAGGAACTTAGTCAT  548648

Query  1206    TGACTGTGGTGGGGCTGCAGGACGATAAGAATTCGGAAGTGAAAGAACTCAGAAGGCAGT  1265
               ||||||||||||||||||||||||||||||||||||||||||||||||||||||||||||
Sbjct  548647  TGACTGTGGTGGGGCTGCAGGACGATAAGAATTCGGAAGTGAAAGAACTCAGAAGGCAGT  548588

Query  1266    GCCAGCAGCTGGAGTCTGTTAAACGGGGCAG--TCTGTGTCAAAAGAGCTTCTCTCCAGA  1323
               ||||||||||||||||| |||||||||||||  |||||||||||||||||||||||||||
Sbjct  548587  GCCAGCAGCTGGAGTCTATTAAACGGGGCAGGATCTGTGTCAAAAGAGCTTCTCTCCAGA  548528

Query  1324    GTAGAAAG  1331
               ||||||||
Sbjct  548527  GTAGAAAG  548520
```

Features flanking this part of subject sequence:
137965 bp at 5' side: magnesium transporter NIPA1 isoform 2
205862 bp at 3' side: putative golgin subfamily A member 6-like protein 6-like ...

Score = 292 bits (158), Expect = 2e-77
Identities = 168/173 (97%), Gaps = 0/173 (0%)
Strand=Plus/Minus

```
Query  678     CAGGAATGCAAAAAGAAATGGAACAGGATGTGAAGAGATTTGGCCAGGCTGCCTGGGCCA  737
               |||||||||| |||||||||||||||||||||||||||||||||||||||||||||||||
Sbjct  554216  CAGGAATGCAGAAAGAAATGGAACAGGATGTGAAGAGATTTGGCCAGGCTGCCTGGGCCA  554157

Query  738     CAGCAATTCCCAGGTTGGAAAAACTTAAGCTAATGCTAGCTCAAGAGACTCTGCAACTCA  797
               ||||||||||||||||||||||||||||||||||||||||||||||||||||||||||||
Sbjct  554156  CAGCAATTCCCAGGTTGGAAAAACTTAAGCTAATGCTAGCTCAAGAGACTCTGCAACTCA  554097

Query  798     TGAGAGCGAAAGAATTGTATTTAAATCGCAAAAGAGCTGAAATTCAGGGAAAG  850
               ||||||||||||| || | |||||||| |||||||||||||||||||||||||
Sbjct  554096  TGAGAGCGAAAGAGTTATGTTTAAATCACAAAAGAGCTGAAATTCAGGGAAAG  554044
```

Features flanking this part of subject sequence:
133890 bp at 5' side: magnesium transporter NIPA1 isoform 2
209978 bp at 3' side: putative golgin subfamily A member 6-like protein 6-like ...

Score = 244 bits (132), Expect = 5e-63
Identities = 132/132 (100%), Gaps = 0/132 (0%)
Strand=Plus/Minus

```
Query  1015    AGATGAAATTTAGAATCAAGTTCAAAACAAAAAACTCTTTGGAATTTTGATTGTGATTTT  1074
               ||||||||||||||||||||||||||||||||||||||||||||||||||||||||||||
Sbjct  550100  AGATGAAATTTAGAATCAAGTTCAAAACAAAAAACTCTTTGGAATTTTGATTGTGATTTT  550041

Query  1075    GCTTAAAATTAGAGATTACTTTGGGGAGAATAGTGGTCTTTGCAATTTTGAATCTTCCTA  1134
               ||||||||||||||||||||||||||||||||||||||||||||||||||||||||||||
Sbjct  550040  GCTTAAAATTAGAGATTACTTTGGGGAGAATAGTGGTCTTTGCAATTTTGAATCTTCCTA  549981

Query  1135    CCCAAGAACATG  1146
               ||||||||||||
Sbjct  549980  CCCAAGAACATG  549969
```

Features flanking this part of subject sequence:
139216 bp at 5' side: magnesium transporter NIPA1 isoform 2
204635 bp at 3' side: putative golgin subfamily A member 6-like protein 6-like ...

Score = 213 bits (115), Expect = 1e-53
Identities = 142/154 (92%), Gaps = 5/154 (3%)
Strand=Plus/Minus

```
Query  528     TCCTTGGATGAGGATGACGTAGGTCCTACGTCTCCAGGATAGTTGCCCTGGAGAAAGAAG  587
               |||||||||||||||||| ||||||||| |     ||| |||||||||||||||||||||
```



```
Sbjct  555443  TCCTTGGATGAGGATGACCTAGGTCCTA-G----AAGGGTAGTTGCCCTGGAGAAAGAAG  555389

Query  588     CTGAAGAATGGACCAGACGGGCTGAAGAAGCTGTTGTCTCTATTCAAGATATCACAGTGA  647
               |||||||||||||||||||||||| ||||||||| |||||||||||||||||||||||||
Sbjct  555388  CTGAAGAATGGACCAGACGGGCTGGAGAAGCTGTCGTCTCTATTCAAGATATCACAGTGA  555329

Query  648     ATTATTTTAATTAGACAGTAAAAGCATTAGCAGG  681
               ||||||||||  ||||||||||||||||||||||
Sbjct  555328  ATTATTTTAAGGAGACAGTAAAAGCATTAGCAGG  555295
```

>**ref|NW_001838191.2|** D Homo sapiens chromosome 15 genomic contig, alternate assembly HuRef SCAF_1103279188272, whole genome shotgun sequence
Length=593693

Sort alignments for this subject sequence by:
E value **Score** **Percent identity**
Query start position **Subject start position**

Features flanking this part of subject sequence:
84076 bp at 3' side: putative golgin subfamily A member 6-like protein 6-like

Score = 329 bits (178), Expect = 1e-88
Identities = 185/188 (98%), Gaps = 2/188 (1%)
Strand=Plus/Plus

```
Query  1146    GAAAAACAGGATGAAGTTGTCTATTACGATCCATGTGAAAGTCCAGAGGAACTTAGTCAT  1205
               ||||||||||||||||||||||||||||||||||||||||||||||||||||||||||||
Sbjct  132656  GAAAAACAGGATGAAGTTGTCTATTACGATCCATGTGAAAGTCCAGAGGAACTTAGTCAT  132715

Query  1206    TGACTGTGGTGGGGCTGCAGGACGATAAGAATTCGGAAGTGAAAGAACTCAGAAGGCAGT  1265
               ||||||||||||||||||||||||||||||||||||||||||||||||||||||||||||
Sbjct  132716  TGACTGTGGTGGGGCTGCAGGACGATAAGAATTCGGAAGTGAAAGAACTCAGAAGGCAGT  132775

Query  1266    GCCAGCAGCTGGAGTCTGTTAAACGGGGCAG--TCTGTGTCAAAAGAGCTTCTCTCCAGA  1323
               ||||||||||||||||| |||||||||||||  |||||||||||||||||||||||||||
Sbjct  132776  GCCAGCAGCTGGAGTCTATTAAACGGGGCAGGATCTGTGTCAAAAGAGCTTCTCTCCAGA  132835

Query  1324    GTAGAAAG  1331
               ||||||||
Sbjct  132836  GTAGAAAG  132843
```

Features flanking this part of subject sequence:
89605 bp at 3' side: putative golgin subfamily A member 6-like protein 6-like

Score = 292 bits (158), Expect = 2e-77
Identities = 168/173 (97%), Gaps = 0/173 (0%)
Strand=Plus/Plus

```
Query  678     CAGGAATGCAAAAAGAAATGGAACAGGATGTGAAGAGATTTGGCCAGGCTGCCTGGGCCA  737
               |||||||||| |||||||||||||||||||||||||||||||||||||||||||||||||
Sbjct  127142  CAGGAATGCAGAAAGAAATGGAACAGGATGTGAAGAGATTTGGCCAGGCTGCCTGGGCCA  127201

Query  738     CAGCAATTCCCAGGTTGGAAAAACTTAAGCTAATGCTAGCTCAAGAGACTCTGCAACTCA  797
               ||||||||||||||||||||||||||||||||||||||||||||||||||||||||||||
Sbjct  127202  CAGCAATTCCCAGGTTGGAAAAACTTAAGCTAATGCTAGCTCAAGAGACTCTGCAACTCA  127261

Query  798     TGAGAGCGAAAGAATTGTATTTAAATCGCAAAAGAGCTGAAATTCAGGGAAAG  850
               ||||||||||||| || | |||||||| |||||||||||||||||||||||||
Sbjct  127262  TGAGAGCGAAAGAGTTATGTTTAAATCACAAAAGAGCTGAAATTCAGGGAAAG  127314
```

Features flanking this part of subject sequence:
85527 bp at 3' side: putative golgin subfamily A member 6-like protein 6-like

Score = 244 bits (132), Expect = 5e-63
Identities = 132/132 (100%), Gaps = 0/132 (0%)
Strand=Plus/Plus

```
Query  1015    AGATGAAATTTAGAATCAAGTTCAAAACAAAAAACTCTTTGGAATTTTGATTGTGATTTT  1074
               ||||||||||||||||||||||||||||||||||||||||||||||||||||||||||||
Sbjct  131261  AGATGAAATTTAGAATCAAGTTCAAAACAAAAAACTCTTTGGAATTTTGATTGTGATTTT  131320

Query  1075    GCTTAAAATTAGAGATTACTTTGGGGAGAATAGTGGTCTTTGCAATTTTGAATCTTCCTA  1134
               ||||||||||||||||||||||||||||||||||||||||||||||||||||||||||||
Sbjct  131321  GCTTAAAATTAGAGATTACTTTGGGGAGAATAGTGGTCTTTGCAATTTTGAATCTTCCTA  131380

Query  1135    CCCAAGAACATG  1146
               ||||||||||||
Sbjct  131381  CCCAAGAACATG  131392
```

Features flanking this part of subject sequence:
90856 bp at 3' side: putative golgin subfamily A member 6-like protein 6-like

Score = 213 bits (115), Expect = 1e-53
Identities = 142/154 (92%), Gaps = 5/154 (3%)
Strand=Plus/Plus

```
Query  528     TCCTTGGATGAGGATGACGTAGGTCCTACGTCTCCAGGATAGTTGCCCTGGAGAAAGAAG  587
               |||||||||||||||||| ||||||||| |     ||| |||||||||||||||||||||
Sbjct  125915  TCCTTGGATGAGGATGACCTAGGTCCTA-G----AAGGGTAGTTGCCCTGGAGAAAGAAG  125969

Query  588     CTGAAGAATGGACCAGACGGGCTGAAGAAGCTGTTGTCTCTATTCAAGATATCACAGTGA  647
               |||||||||||||||||||||||| ||||||||| |||||||||||||||||||||||||
Sbjct  125970  CTGAAGAATGGACCAGACGGGCTGGAGAAGCTGTCGTCTCTATTCAAGATATCACAGTGA  126029

Query  648     ATTATTTTAATTAGACAGTAAAAGCATTAGCAGG  681
               ||||||||||  ||||||||||||||||||||||
Sbjct  126030  ATTATTTTAAGGAGACAGTAAAAGCATTAGCAGG  126063
```

>ref|NT_026446.14| D Homo sapiens chromosome 15 genomic contig, GRCh37.p5 Primary Assembly
Length=5594590

Sort alignments for this subject sequence by:
E value **Score** **Percent identity**
Query start position **Subject start position**

Features flanking this part of subject sequence:
43507 bp at 5' side: putative golgin subfamily A member 8I

Score = 329 bits (178), Expect = 1e-88
Identities = 185/188 (98%), Gaps = 2/188 (1%)
Strand=Plus/Plus

```
Query  1146     GAAAAACAGGATGAAGTTGTCTATTACGATCCATGTGAAAGTCCAGAGGAACTTAGTCAT  1205
                ||||||||||||||||||||||||||||||||||||||||||||||||||||||||||||
Sbjct  5431780  GAAAAACAGGATGAAGTTGTCTATTACGATCCATGTGAAAGTCCAGAGGAACTTAGTCAT  5431839

Query  1206     TGACTGTGGTGGGGCTGCAGGACGATAAGAATTCGGAAGTGAAAGAACTCAGAAGGCAGT  1265
                ||||||||||||||||||||||||||||||||||||||||||||||||||||||||||||
Sbjct  5431840  TGACTGTGGTGGGGCTGCAGGACGATAAGAATTCGGAAGTGAAAGAACTCAGAAGGCAGT  5431899

Query  1266     GCCAGCAGCTGGAGTCTGTTAAACGGGGCAG--TCTGTGTCAAAAGAGCTTCTCTCCAGA  1323
                ||||||||||||||||| |||||||||||||  |||||||||||||||||||||||||||
Sbjct  5431900  GCCAGCAGCTGGAGTCTATTAAACGGGGCAGGATCTGTGTCAAAAGAGCTTCTCTCCAGA  5431959

Query  1324     GTAGAAAG  1331
                ||||||||
Sbjct  5431960  GTAGAAAG  5431967
```

Features flanking this part of subject sequence:
37997 bp at 5' side: putative golgin subfamily A member 8I

Score = 309 bits (167), Expect = 2e-82
Identities = 171/173 (99%), Gaps = 0/173 (0%)
Strand=Plus/Plus

```
Query  678      CAGGAATGCAAAAAGAAATGGAACAGGATGTGAAGAGATTTGGCCAGGCTGCCTGGGCCA  737
                |||||||||||||||||||||||||||||||||||||| |||||||||||||||||||||
Sbjct  5426270  CAGGAATGCAAAAAGAAATGGAACAGGATGTGAAGAGACTTGGCCAGGCTGCCTGGGCCA  5426329

Query  738      CAGCAATTCCCAGGTTGGAAAAACTTAAGCTAATGCTAGCTCAAGAGACTCTGCAACTCA  797
                ||||||||||||||||||||||||||||||||||||||||||||||||||||||||||||
Sbjct  5426330  CAGCAATTCCCAGGTTGGAAAAACTTAAGCTAATGCTAGCTCAAGAGACTCTGCAACTCA  5426389

Query  798      TGAGAGCGAAAGAATTGTATTTAAATCGCAAAAGAGCTGAAATTCAGGGAAAG  850
                |||||||||||||||||||||||||| ||||||||||||||||||||||||||
Sbjct  5426390  TGAGAGCGAAAGAATTGTATTTAAATTGCAAAAGAGCTGAAATTCAGGGAAAG  5426442
```

Features flanking this part of subject sequence:
42113 bp at 5' side: putative golgin subfamily A member 8I

Score = 239 bits (129), Expect = 2e-61
Identities = 129/129 (100%), Gaps = 0/129 (0%)
Strand=Plus/Plus

```
Query  1015     AGATGAAATTTAGAATCAAGTTCAAAACAAAAAACTCTTTGGAATTTTGATTGTGATTTT  1074
                ||||||||||||||||||||||||||||||||||||||||||||||||||||||||||||
Sbjct  5430386  AGATGAAATTTAGAATCAAGTTCAAAACAAAAAACTCTTTGGAATTTTGATTGTGATTTT  5430445

Query  1075     GCTTAAAATTAGAGATTACTTTGGGGAGAATAGTGGTCTTTGCAATTTTGAATCTTCCTA  1134
                ||||||||||||||||||||||||||||||||||||||||||||||||||||||||||||
Sbjct  5430446  GCTTAAAATTAGAGATTACTTTGGGGAGAATAGTGGTCTTTGCAATTTTGAATCTTCCTA  5430505

Query  1135     CCCAAGAAC  1143
                |||||||||
Sbjct  5430506  CCCAAGAAC  5430514
```

Features flanking this part of subject sequence:
36772 bp at 5' side: putative golgin subfamily A member 8I

Score = 213 bits (115), Expect = 1e-53
Identities = 142/154 (92%), Gaps = 5/154 (3%)
Strand=Plus/Plus

```
Query  528      TCCTTGGATGAGGATGACGTAGGTCCTACGTCTCCAGGATAGTTGCCCTGGAGAAAGAAG  587
                |||||||||||||||||| ||||||||| |     ||| |||||||||||||||||||||
Sbjct  5425045  TCCTTGGATGAGGATGACCTAGGTCCTA-G----AAGGGTAGTTGCCCTGGAGAAAGAAG  5425099

Query  588      CTGAAGAATGGACCAGACGGGCTGAAGAAGCTGTTGTCTCTATTCAAGATATCACAGTGA  647
                |||||||||||||||||||||||| ||||||||| |||||||||||||||||||||||||
Sbjct  5425100  CTGAAGAATGGACCAGACGGGCTGGAGAAGCTGTCGTCTCTATTCAAGATATCACAGTGA  5425159

Query  648      ATTATTTTAATTAGACAGTAAAAGCATTAGCAGG  681
                ||||||||||  ||||||||||||||||||||||
Sbjct  5425160  ATTATTTTAAGGAGACAGTAAAAGCATTAGCAGG  5425193
```

>ref|NW_001838221.1| D Homo sapiens chromosome 15 genomic contig, alternate assembly HuRef SCAF_1103279188173, whole genome shotgun sequence
Length=1688394

Sort alignments for this subject sequence by:

E value **Score** **Percent identity**
Query start position **Subject start position**

Features in this part of subject sequence:
WASP homolog-associated protein with actin, membranes and...

Score = 303 bits (164), Expect = 8e-81
Identities = 174/179 (97%), Gaps = 0/179 (0%)
Strand=Plus/Plus

```
Query  848     AAGATGGAAGATCTTCCAGAACAAGAAAAAAATATAAATGTTGTAGATGAATTAGAAATG  907
               |||||||||||||||||||||||||||||||||| |||||||||||||||||||||||| 
Sbjct  298126  AAGATGGAAGATCTTCCAGAACAAGAAAAAAATACAAATGTTGTAGATGAATTAGAAATA  298185

Query  908     CAATTTTATGAAATTCAGTTAGAACTATATGAAGTTAAATTTGAGATATTAAAAAACGAA  967
               ||||||||||||||||| ||||||||||||||||||||||||||||||||||||||||||
Sbjct  298186  CAATTTTATGAAATTCAATTAGAACTATATGAAGTTAAATTTGAGATATTAAAAAACGAA  298245

Query  968     GAAATACTGCTTACTACACAGTTGGACTCTCTTAAAAGACTTATAAAAGATGAAATTTA  1026
               ||||||||||||||||||||||||||||||||||||||||||||||||| | |||||||
Sbjct  298246  GAAATACTGCTTACTACACAGTTGGACTCTCTTAAAAGACTTATAAAAGGTAAAATTTA  298304
```

Features in this part of subject sequence:
WASP homolog-associated protein with actin, membranes and...

Score = 276 bits (149), Expect = 2e-72
Identities = 165/173 (95%), Gaps = 0/173 (0%)
Strand=Plus/Plus

```
Query  678     CAGGAATGCAAAAAGAAATGGAACAGGATGTGAAGAGATTTGGCCAGGCTGCCTGGGCCA  737
               |||||||||| ||||||||||||||||||| |||||||||||| ||||||||||||||||
Sbjct  296723  CAGGAATGCAGAAAGAAATGGAACAGGATGCGAAGAGATTTGGTCAGGCTGCCTGGGCCA  296782

Query  738     CAGCAATTCCCAGGTTGGAAAAACTTAAGCTAATGCTAGCTCAAGAGACTCTGCAACTCA  797
               |||||||||||||||||||||||||| ||||||||||||||| |||||||||||||||||
Sbjct  296783  CAGCAATTCCCAGGTTGGAAAAACTTCAGCTAATGCTAGCTCGAGAGACTCTGCAACTCA  296842

Query  798     TGAGAGCGAAAGAATTGTATTTAAATCGCAAAAGAGCTGAAATTCAGGGAAAG  850
               ||||||||||||| |||| |||||||| |||||||||||||||||||||||||
Sbjct  296843  TGAGAGCGAAAGAGTTGTGTTTAAATCACAAAAGAGCTGAAATTCAGGGAAAG  296895
```

Features in this part of subject sequence:
WASP homolog-associated protein with actin, membranes and...

Score = 257 bits (139), Expect = 6e-67
Identities = 176/192 (92%), Gaps = 9/192 (5%)
Strand=Plus/Plus

```
Query  1146    GAAAAACAGGATGAAGTTGTCTATTACGATCCATGTGAAAGTCCAGAGGAACTT--AGTC  1203
               |||||||| ||||||||||||||||||||||||||||||| |||||||||||||  ||||
Sbjct  301892  GAAAAACAAGATGAAGTTGTCTATTACGATCCATGTGAAAATCCAGAGGAACTTAAAGTC  301951

Query  1204    ATTGAC--TGTGGTGGGGCTGCAGGACGATAAGAATTCGGAAGTGAAAGAACTCAGAAGG  1261
               ||||||  |||||||||||||||||| |||||||||| ||||||||||||||||||||||
Sbjct  301952  ATTGACTGTGTGGTGGGGCTGCAGGATGATAAGAATTTGGAAGTGAAAGAACTCAGAAGG  302011

Query  1262    CAGTGCCAGCAGCTGGAGTCTGTTAAACGGGGCAG--TCTGTGTCAAAAGAGCTTCTCTC  1319
               |||||||||||||||||||||   |||||||||||  |||||| ||||||||| ||||||
Sbjct  302012  CAGTGCCAGCAGCTGGAGTCT---AAACGGGGCAGGATCTGTGCCAAAAGAGCCTCTCTC  302068

Query  1320    CAGAGTAGAAAG  1331
               | ||||||||||
Sbjct  302069  CGGAGTAGAAAG  302080
```

Features in this part of subject sequence:
WASP homolog-associated protein with actin, membranes and...

Score = 231 bits (125), Expect = 4e-59
Identities = 130/132 (98%), Gaps = 1/132 (1%)
Strand=Plus/Plus

```
Query  1015    AGATGAAATTTAGAATCAAGTTCAAAACAAAAAACTCTTTGGAATTTTGATTGTGATTTT  1074
               |||||||||||||||||||||||||||| |||||||| ||||||||||||||||||||||
Sbjct  300502  AGATGAAATTTAGAATCAAGTTCAAAAC-AAAAACTCGTTGGAATTTTGATTGTGATTTT  300560

Query  1075    GCTTAAAATTAGAGATTACTTTGGGGAGAATAGTGGTCTTTGCAATTTTGAATCTTCCTA  1134
               ||||||||||||||||||||||||||||||||||||||||||||||||||||||||||||
Sbjct  300561  GCTTAAAATTAGAGATTACTTTGGGGAGAATAGTGGTCTTTGCAATTTTGAATCTTCCTA  300620

Query  1135    CCCAAGAACATG  1146
               ||||||||||||
Sbjct  300621  CCCAAGAACATG  300632
```

Features in this part of subject sequence:
WASP homolog-associated protein with actin, membranes and...

Score = 207 bits (112), Expect = 6e-52
Identities = 139/151 (92%), Gaps = 5/151 (3%)
Strand=Plus/Plus

```
Query  531     TTGGATGAGGATGACGTAGGTCCTACGTCTCCAGGATAGTTGCCCTGGAGAAAGAAGCTG  590
               ||||||||||||||| ||||||||| |     ||| ||||||||||||||||||||||||
Sbjct  295504  TTGGATGAGGATGACCTAGGTCCTA-G----AAGGGTAGTTGCCCTGGAGAAAGAAGCTG  295558

Query  591     AAGAATGGACCAGACGGGCTGAAGAAGCTGTTGTCTCTATTCAAGATATCACAGTGAATT  650
               ||||||||||||||||||||||||||||||| ||||||||||| ||||||||||||||||
Sbjct  295559  AAGAATGGACCAGACGGGCTGAAGAAGCTGTCGTCTCTATTCAGGATATCACAGTGAATT  295618

Query  651     ATTTTAATTAGACAGTAAAAGCATTAGCAGG  681
```

```
                ||||||  ||||||||||||||||||||||
Sbjct  295619  ATTTTAAGGAGACAGTAAAAGCATTAGCAGG  295649
```

>ref|NT_077661.3| D Homo sapiens chromosome 15 genomic contig, GRCh37.p5 Primary Assembly
Length=2104828

Sort alignments for this subject sequence by:
E value **Score** **Percent identity**
Query start position **Subject start position**

Features in this part of subject sequence:
WASP homolog-associated protein with actin, membranes and...

Score = 303 bits (164), Expect = 8e-81
Identities = 174/179 (97%), Gaps = 0/179 (0%)
Strand=Plus/Plus

```
Query  848     AAGATGGAAGATCTTCCAGAACAAGAAAAAAATATAAATGTTGTAGATGAATTAGAAATG  907
               |||||||||||||||||||||||||||||||||| ||||||||||||||||||||||||
Sbjct  608427  AAGATGGAAGATCTTCCAGAACAAGAAAAAAATACAAATGTTGTAGATGAATTAGAAATA  608486

Query  908     CAATTTTATGAAATTCAGTTAGAACTATATGAAGTTAAATTTGAGATATTAAAAAACGAA  967
               ||||||||||||||||| ||||||||||||||||||||||||||||||||||||||||||
Sbjct  608487  CAATTTTATGAAATTCAATTAGAACTATATGAAGTTAAATTTGAGATATTAAAAAACGAA  608546

Query  968     GAAATACTGCTTACTACACAGTTGGACTCTCTTAAAAGACTTATAAAAGATGAAATTTA  1026
               ||||||||||||||||||||||||||||||||||||||||||||||||| | |||||||
Sbjct  608547  GAAATACTGCTTACTACACAGTTGGACTCTCTTAAAAGACTTATAAAAGGTAAAATTTA  608605
```

Features in this part of subject sequence:
WASP homolog-associated protein with actin, membranes and...

Score = 276 bits (149), Expect = 2e-72
Identities = 165/173 (95%), Gaps = 0/173 (0%)
Strand=Plus/Plus

```
Query  678     CAGGAATGCAAAAAGAAATGGAACAGGATGTGAAGAGATTTGGCCAGGCTGCCTGGGCCA  737
               |||||||||| ||||||||||||||||||| |||||||||||| ||||||||||||||||
Sbjct  607024  CAGGAATGCAGAAAGAAATGGAACAGGATGCGAAGAGATTTGGTCAGGCTGCCTGGGCCA  607083

Query  738     CAGCAATTCCCAGGTTGGAAAAACTTAAGCTAATGCTAGCTCAAGAGACTCTGCAACTCA  797
               |||||||||||||||||||||||||| ||||||||||||||| |||||||||||||||||
Sbjct  607084  CAGCAATTCCCAGGTTGGAAAAACTTCAGCTAATGCTAGCTCGAGAGACTCTGCAACTCA  607143

Query  798     TGAGAGCGAAAGAATTGTATTTAAATCGCAAAAGAGCTGAAATTCAGGGAAAG  850
               ||||||||||||| |||| |||||||| |||||||||||||||||||||||||
Sbjct  607144  TGAGAGCGAAAGAGTTGTGTTTAAATCACAAAAGAGCTGAAATTCAGGGAAAG  607196
```

Features in this part of subject sequence:
WASP homolog-associated protein with actin, membranes and...

Score = 257 bits (139), Expect = 6e-67
Identities = 176/192 (92%), Gaps = 9/192 (5%)
Strand=Plus/Plus

```
Query  1146    GAAAAACAGGATGAAGTTGTCTATTACGATCCATGTGAAAGTCCAGAGGAACTT--AGTC  1203
               |||||||| ||||||||||||||||||||||||||||||| |||||||||||||  ||||
Sbjct  612206  GAAAAACAAGATGAAGTTGTCTATTACGATCCATGTGAAAATCCAGAGGAACTTAAAGTC  612265

Query  1204    ATTGAC--TGTGGTGGGGCTGCAGGACGATAAGAATTCGGAAGTGAAAGAACTCAGAAGG  1261
               ||||||  |||||||||||||||||| |||||||||| ||||||||||||||||||||||
Sbjct  612266  ATTGACTGTGTGGTGGGGCTGCAGGATGATAAGAATTTGGAAGTGAAAGAACTCAGAAGG  612325

Query  1262    CAGTGCCAGCAGCTGGAGTCTGTTAAACGGGGCAG--TCTGTGTCAAAAGAGCTTCTCTC  1319
               |||||||||||||||||||||   |||||||||||  |||||| ||||||||| ||||||
Sbjct  612326  CAGTGCCAGCAGCTGGAGTCT---AAACGGGGCAGGATCTGTGCCAAAAGAGCCTCTCTC  612382

Query  1320    CAGAGTAGAAAG  1331
               | ||||||||||
Sbjct  612383  CGGAGTAGAAAG  612394
```

Features in this part of subject sequence:
WASP homolog-associated protein with actin, membranes and...

Score = 231 bits (125), Expect = 4e-59
Identities = 130/132 (98%), Gaps = 1/132 (1%)
Strand=Plus/Plus

```
Query  1015    AGATGAAATTTAGAATCAAGTTCAAAACAAAAAACTCTTTGGAATTTTGATTGTGATTTT  1074
               |||||||||||||||||||||||||||| |||||||| ||||||||||||||||||||||
Sbjct  610816  AGATGAAATTTAGAATCAAGTTCAAAAC-AAAAACTCGTTGGAATTTTGATTGTGATTTT  610874

Query  1075    GCTTAAAATTAGAGATTACTTTGGGGAGAATAGTGGTCTTTGCAATTTTGAATCTTCCTA  1134
               ||||||||||||||||||||||||||||||||||||||||||||||||||||||||||||
Sbjct  610875  GCTTAAAATTAGAGATTACTTTGGGGAGAATAGTGGTCTTTGCAATTTTGAATCTTCCTA  610934

Query  1135    CCCAAGAACATG  1146
               ||||||||||||
Sbjct  610935  CCCAAGAACATG  610946
```

Features in this part of subject sequence:
WASP homolog-associated protein with actin, membranes and...

Score = 207 bits (112), Expect = 6e-52

Identities = 139/151 (92%), Gaps = 5/151 (3%)
Strand=Plus/Plus

```
Query  531     TTGGATGAGGATGACGTAGGTCCTACGTCTCCAGGATAGTTGCCCTGGAGAAAGAAGCTG  590
               ||||||||||||||| ||||||||| |     ||| ||||||||||||||||||||||||
Sbjct  605805  TTGGATGAGGATGACCTAGGTCCTA-G----AAGGGTAGTTGCCCTGGAGAAAGAAGCTG  605859

Query  591     AAGAATGGACCAGACGGGCTGAAGAAGCTGTTGTCTCTATTCAAGATATCACAGTGAATT  650
               ||||||||||||||||||||||||||||||| ||||||||||| ||||||||||||||||
Sbjct  605860  AAGAATGGACCAGACGGGCTGAAGAAGCTGTCGTCTCTATTCAGGATATCACAGTGAATT  605919

Query  651     ATTTTAATTAGACAGTAAAAGCATTAGCAGG  681
               |||||||  ||||||||||||||||||||||
Sbjct  605920  ATTTTAAGGAGACAGTAAAAGCATTAGCAGG  605950
```

>ref|XR_132589.1| GM PREDICTED: Homo sapiens u3 small nucleolar ribonucleoprotein protein MPP10-like (LOC100652840), miscRNA
Length=2207

GENE ID: 100652840 LOC100652840 | u3 small nucleolar ribonucleoprotein protein MPP10-like [Homo sapiens]

Score = 676 bits (366), Expect = 0.0
Identities = 370/372 (99%), Gaps = 0/372 (0%)
Strand=Plus/Plus

```
Query  1189  CAGAGGAACTTAGTCATTGACTGTGGTGGGGCTGCAGGACGATAAGAATTCGGAAGTGAA  1248
             ||||||||||||||||||||||||||||||||||||||||||||||||||||||||||||
Sbjct  1     CAGAGGAACTTAGTCATTGACTGTGGTGGGGCTGCAGGACGATAAGAATTCGGAAGTGAA  60

Query  1249  AGAACTCAGAAGGCAGTGCCAGCAGCTGGAGTCTGTTAAACGGGGCAGTCTGTGTCAAAA  1308
             ||||||||||||||||||||||||||||||||||||||||||| ||||||||||||||||
Sbjct  61    AGAACTCAGAAGGCAGTGCCAGCAGCTGGAGTCTGTTAAACGGAGCAGTCTGTGTCAAAA  120

Query  1309  GAGCTTCTCTCCAGAGTAGAAAGATTTATTTTCTTTTTGAATATATTTTCTACACCATGG  1368
             ||||||||||||||||||||||||||||||||||||||||||||||||||||||||||||
Sbjct  121   GAGCTTCTCTCCAGAGTAGAAAGATTTATTTTCTTTTTGAATATATTTTCTACACCATGG  180

Query  1369  TTTCTCAACCAGAGGTGATACAGCACTGTCTCCAAATGTGGGGCTGTTGTGTATTGTCAC  1428
             ||||||||||||||||||||||||||||||||||||||||| ||||||||||||||||||
Sbjct  181   TTTCTCAACCAGAGGTGATACAGCACTGTCTCCAAATGTGGAGCTGTTGTGTATTGTCAC  240

Query  1429  GGGGAGGAGAAATGCTACTGGCATTTAGTGGATGATGGTCAAGGACACTAAGCAACCTGC  1488
             ||||||||||||||||||||||||||||||||||||||||||||||||||||||||||||
Sbjct  241   GGGGAGGAGAAATGCTACTGGCATTTAGTGGATGATGGTCAAGGACACTAAGCAACCTGC  300

Query  1489  AATGTGTGGATCAATCTTACACAACGAAGGACTGTTCCACCCCAAATGCCAATGGTACCC  1548
             ||||||||||||||||||||||||||||||||||||||||||||||||||||||||||||
Sbjct  301   AATGTGTGGATCAATCTTACACAACGAAGGACTGTTCCACCCCAAATGCCAATGGTACCC  360

Query  1549  TGTTGAGAAAGG  1560
             ||||||||||||
Sbjct  361   TGTTGAGAAAGG  372
```

>ref|NR_026589.1| EGM Homo sapiens WAS protein homolog associated with actin, golgi membranes and microtubules pseudogene 2 (WHAMMP2), non-coding RNA
Length=5246

GENE ID: 440253 WHAMMP2 | WAS protein homolog associated with actin, golgi membranes and microtubules pseudogene 2 [Homo sapiens]
(10 or fewer PubMed links)

Sort alignments for this subject sequence by:
E value **Score** **Percent identity**
Query start position **Subject start position**

Score = 599 bits (324), Expect = 9e-170
Identities = 334/339 (99%), Gaps = 0/339 (0%)
Strand=Plus/Plus

```
Query  679   AGGAATGCAAAAAGAAATGGAACAGGATGTGAAGAGATTTGGCCAGGCTGCCTGGGCCAC  738
             ||||||||||||||||||||||||||||||||||||| ||||||||||||||||||||||
Sbjct  1086  AGGAATGCAAAAAGAAATGGAACAGGATGTGAAGAGACTTGGCCAGGCTGCCTGGGCCAC  1145

Query  739   AGCAATTCCCAGGTTGGAAAAACTTAAGCTAATGCTAGCTCAAGAGACTCTGCAACTCAT  798
             ||||||||||||||||||||||||||||||||||||||||||||||||||||||||||||
Sbjct  1146  AGCAATTCCCAGGTTGGAAAAACTTAAGCTAATGCTAGCTCAAGAGACTCTGCAACTCAT  1205

Query  799   GAGAGCGAAAGAATTGTATTTAAATCGCAAAAGAGCTGAAATTCAGGGAAAGATGGAAGA  858
             ||||||||||||||||||||||||| ||||||||||||||||||||||||||||||||||
Sbjct  1206  GAGAGCGAAAGAATTGTATTTAAATTGCAAAAGAGCTGAAATTCAGGGAAAGATGGAAGA  1265

Query  859   TCTTCCAGAACAAGAAAAAAATATAAATGTTGTAGATGAATTAGAAATGCAATTTTATGA  918
             |||||||||||||||||||||||||||||||||||||||||||||||| |||||||||||
Sbjct  1266  TCTTCCAGAACAAGAAAAAAATATAAATGTTGTAGATGAATTAGAAATACAATTTTATGA  1325

Query  919   AATTCAGTTAGAACTATATGAAGTTAAATTTGAGATATTAAAAAACGAAGAAATACTGCT  978
             |||||||||||||||||||||||||||||||||||||||||||||| |||||||||||||
Sbjct  1326  AATTCAGTTAGAACTATATGAAGTTAAATTTGAGATATTAAAAAACAAAGAAATACTGCT  1385

Query  979   TACTACACAGTTGGACTCTCTTAAAAGACTTATAAAAGA  1017
             |||||||||||||||||||||| ||||||||||||||||
Sbjct  1386  TACTACACAGTTGGACTCTCTTGAAAGACTTATAAAAGA  1424
```

Score = 398 bits (215), Expect = 3e-109
Identities = 298/334 (89%), Gaps = 22/334 (7%)
Strand=Plus/Plus

```
Query  366  AGGTTATCCAAGGACATGGAAAAGCCAACACCATGGTAGCATTAATGAAAGTTTACCAAG  425
            ||||||| |||||||| |||||||||||||||||||||||||||||||||||||||||||
Sbjct  602  AGGTTATTCAAGGACACGGAAAAGCCAACACCATGGTAGCATTAATGAAAGTTTACCAAG  661

Query  426  AGGAAGATGAAGCCTACCAGGAATTAGTTACCATGGCAACCATGTTTTTCCAGTACTTAC  485
            |||||||||||||||||||||||||||||||| ||||||||| ||| |||||||||||| 
Sbjct  662  AGGAAGATGAAGCCTACCAGGAATTAGTTACCGTGGCAACCACGTTCTTCCAGTACTTAT  721

Query  486  TGCAGCCATTTAGGGCTATGCGAGAAGTTGCAACTTTAT--AA--TT------------  528
            ||| |||||||||||||||||||||||||||||||||||  ||  ||
Sbjct  722  TGCGGCCATTTAGGGCTATGCGAGAAGTTGCAACTTTATGTAAGCTTGATATTTTGAAGT  781

Query  529  CCTTGGATGAGGATGACGTAGGTCCTACGTCTCCAGGATAGTTGCCCTGGAGAAAGAAGC  588
            ||||||||||||||||| ||||||||| |     ||| ||||||||||||||||||||||
Sbjct  782  CCTTGGATGAGGATGACCTAGGTCCTA-GA----AGGGTAGTTGCCCTGGAGAAAGAAGC  836

Query  589  TGAAGAATGGACCAGACGGGCTGAAGAAGCTGTTGTCTCTATTCAAGATATCACAGTGAA  648
            ||||||||||||||||||||||| ||||||||| ||||||||||||||||||||||||||
Sbjct  837  TGAAGAATGGACCAGACGGGCTGGAGAAGCTGTCGTCTCTATTCAAGATATCACAGTGAA  896

Query  649  TTATTTTAATTAGACAGTAAAAGCATTAGCAGGA  682
            |||||||||  |||||||||||||||||||||||
Sbjct  897  TTATTTTAAGGAGACAGTAAAAGCATTAGCAGGA  930
```

```
 Score =  329 bits (178),  Expect = 1e-88
 Identities = 185/188 (98%), Gaps = 2/188 (1%)
 Strand=Plus/Plus

Query  1146  GAAAAACAGGATGAAGTTGTCTATTACGATCCATGTGAAAGTCCAGAGGAACTTAGTCAT  1205
             ||||||||||||||||||||||||||||||||||||||||||||||||||||||||||||
Sbjct  1423  GAAAAACAGGATGAAGTTGTCTATTACGATCCATGTGAAAGTCCAGAGGAACTTAGTCAT  1482

Query  1206  TGACTGTGGTGGGGCTGCAGGACGATAAGAATTCGGAAGTGAAAGAACTCAGAAGGCAGT  1265
             ||||||||||||||||||||||||||||||||||||||||||||||||||||||||||||
Sbjct  1483  TGACTGTGGTGGGGCTGCAGGACGATAAGAATTCGGAAGTGAAAGAACTCAGAAGGCAGT  1542

Query  1266  GCCAGCAGCTGGAGTCTGTTAAACGGGGCAG--TCTGTGTCAAAAGAGCTTCTCTCCAGA  1323
             ||||||||||||||||| |||||||||||||  |||||||||||||||||||||||||||
Sbjct  1543  GCCAGCAGCTGGAGTCTATTAAACGGGGCAGGATCTGTGTCAAAAGAGCTTCTCTCCAGA  1602

Query  1324  GTAGAAAG  1331
             ||||||||
Sbjct  1603  GTAGAAAG  1610
```

>**ref|NW_001838205.1|** G Homo sapiens chromosome 15 genomic contig, alternate assembly
HuRef SCAF_1103279183344, whole genome shotgun sequence
Length=8319

GENE ID: 100288615 LOC100288615 | WAS protein homolog associated with actin,
golgi membranes and microtubules pseudogene [Homo sapiens]
(10 or fewer PubMed links)

Sort alignments for this subject sequence by:
E value **Score** **Percent identity**
Query start position **Subject start position**

```
 Score =  315 bits (170),  Expect = 4e-84
 Identities = 177/180 (98%), Gaps = 2/180 (1%)
 Strand=Plus/Plus

Query  848   AAGATGGAAGATCTTCCAGAACAAGAAAAAAATATAAATGTTGTAGATGAATTAGAAATG  907
             ||||||||||||||||||||||||||||||||||||||||||||||||||||||||||||
Sbjct  5527  AAGATGGAAGATCTTCCAGAACAAGAAAAAAATATAAATGTTGTAGATGAATTAGAAATG  5586

Query  908   CAATTTTATGAAATTCAGTTAGAACTATATGAAGTTAAATTTGAGATATTAAAAAACGAA  967
             ||||||||||||||||||||||||||||||||||||||||||||||||||||||||||||
Sbjct  5587  CAATTTTATGAAATTCAGTTAGAACTATATGAAGTTAAATTTGAGATATTAAAAAACGAA  5646

Query  968   GAAATACTGCTTACTACACAGTTGGACTCTCTTAAAAGACTTATAAAAGATGAAA-TTTA  1026
             ||||||||||||||||||||||||||||||||||||||||||||||||| | ||| ||||
Sbjct  5647  GAAATACTGCTTACTACACAGTTGGACTCTCTTAAAAGACTTATAAAAGGT-AAAGTTTA  5705
```

```
 Score =  291 bits (157),  Expect = 6e-77
 Identities = 157/157 (100%), Gaps = 0/157 (0%)
 Strand=Plus/Plus

Query  525   AATTCCTTGGATGAGGATGACGTAGGTCCTACGTCTCCAGGATAGTTGCCCTGGAGAAAG  584
             ||||||||||||||||||||||||||||||||||||||||||||||||||||||||||||
Sbjct  2570  AATTCCTTGGATGAGGATGACGTAGGTCCTACGTCTCCAGGATAGTTGCCCTGGAGAAAG  2629

Query  585   AAGCTGAAGAATGGACCAGACGGGCTGAAGAAGCTGTTGTCTCTATTCAAGATATCACAG  644
             ||||||||||||||||||||||||||||||||||||||||||||||||||||||||||||
Sbjct  2630  AAGCTGAAGAATGGACCAGACGGGCTGAAGAAGCTGTTGTCTCTATTCAAGATATCACAG  2689

Query  645   TGAATTATTTTAATTAGACAGTAAAAGCATTAGCAGG  681
             |||||||||||||||||||||||||||||||||||||
Sbjct  2690  TGAATTATTTTAATTAGACAGTAAAAGCATTAGCAGG  2726
```

```
 Score =  244 bits (132),  Expect = 5e-63
 Identities = 132/132 (100%), Gaps = 0/132 (0%)
 Strand=Plus/Plus

Query  1015  AGATGAAATTTAGAATCAAGTTCAAAACAAAAAACTCTTTGGAATTTTGATTGTGATTTT  1074
             ||||||||||||||||||||||||||||||||||||||||||||||||||||||||||||
Sbjct  7905  AGATGAAATTTAGAATCAAGTTCAAAACAAAAAACTCTTTGGAATTTTGATTGTGATTTT  7964

Query  1075  GCTTAAAATTAGAGATTACTTTGGGGAGAATAGTGGTCTTTGCAATTTTGAATCTTCCTA  1134
```



```
             ||||||||||||||||||||||||||||||||||||||||||||||||||||||||||||
Sbjct  7965  GCTTAAAATTAGAGATTACTTTGGGGAGAATAGTGGTCTTTGCAATTTTGAATCTTCCTA  8024

Query  1135  CCCAAGAACATG  1146
             ||||||||||||
Sbjct  8025  CCCAAGAACATG  8036
```

>**ref|NT_010194.17|** D Homo sapiens chromosome 15 genomic contig, GRCh37.p5 Primary
Assembly
Length=53620202

Sort alignments for this subject sequence by:
E value **Score** **Percent identity**
Query start position **Subject start position**

Features flanking this part of subject sequence:
59827 bp at 5' side: golgin subfamily A member 8-like protein 2-like
129614 bp at 3' side: golgin subfamily A member 8H isoform 1

```
 Score =  968 bits (524),  Expect = 0.0
 Identities = 524/524 (100%), Gaps = 0/524 (0%)
 Strand=Plus/Plus

Query  1        GTTGGTCAGGCTGGTGTCGAAGTCCTGACCTCAAGTGATCCACCCGCCTCGGCCTCCCAA  60
                ||||||||||||||||||||||||||||||||||||||||||||||||||||||||||||
Sbjct  1556749  GTTGGTCAGGCTGGTGTCGAAGTCCTGACCTCAAGTGATCCACCCGCCTCGGCCTCCCAA  1556808

Query  61       AGTGCTGGAATTACAGGCGTGAGCCACGGTGCCTGGCCAAAAGTCTCCATATTATTAAAC  120
                ||||||||||||||||||||||||||||||||||||||||||||||||||||||||||||
Sbjct  1556809  AGTGCTGGAATTACAGGCGTGAGCCACGGTGCCTGGCCAAAAGTCTCCATATTATTAAAC  1556868

Query  121      AATCTTCAGAAGCACAGTGCTGAATGACTACAGTAATAATATTCTGCCATGGATATATCA  180
                ||||||||||||||||||||||||||||||||||||||||||||||||||||||||||||
Sbjct  1556869  AATCTTCAGAAGCACAGTGCTGAATGACTACAGTAATAATATTCTGCCATGGATATATCA  1556928

Query  181      TAATTTACTTAACAATTCTTGTTTTATTGGGCATTTTTGATGGAGGATGATGACATTTTC  240
                ||||||||||||||||||||||||||||||||||||||||||||||||||||||||||||
Sbjct  1556929  TAATTTACTTAACAATTCTTGTTTTATTGGGCATTTTTGATGGAGGATGATGACATTTTC  1556988

Query  241      ATATTTAATCAATATTTTAAATTGATGTATTGAAAGTTGAGAACATGAAGGTTTCTTTTG  300
                ||||||||||||||||||||||||||||||||||||||||||||||||||||||||||||
Sbjct  1556989  ATATTTAATCAATATTTTAAATTGATGTATTGAAAGTTGAGAACATGAAGGTTTCTTTTG  1557048

Query  301      TTTAGTTTTGTTTGTTGGGTATGTATTACACTGTCTGACTTGAGCTTTATTCACATTTGC  360
                ||||||||||||||||||||||||||||||||||||||||||||||||||||||||||||
Sbjct  1557049  TTTAGTTTTGTTTGTTGGGTATGTATTACACTGTCTGACTTGAGCTTTATTCACATTTGC  1557108

Query  361      TCTCTAGGTTATCCAAGGACATGGAAAAGCCAACACCATGGTAGCATTAATGAAAGTTTA  420
                ||||||||||||||||||||||||||||||||||||||||||||||||||||||||||||
Sbjct  1557109  TCTCTAGGTTATCCAAGGACATGGAAAAGCCAACACCATGGTAGCATTAATGAAAGTTTA  1557168

Query  421      CCAAGAGGAAGATGAAGCCTACCAGGAATTAGTTACCATGGCAACCATGTTTTTCCAGTA  480
                ||||||||||||||||||||||||||||||||||||||||||||||||||||||||||||
Sbjct  1557169  CCAAGAGGAAGATGAAGCCTACCAGGAATTAGTTACCATGGCAACCATGTTTTTCCAGTA  1557228

Query  481      CTTACTGCAGCCATTTAGGGCTATGCGAGAAGTTGCAACTTTAT  524
                ||||||||||||||||||||||||||||||||||||||||||||
Sbjct  1557229  CTTACTGCAGCCATTTAGGGCTATGCGAGAAGTTGCAACTTTAT  1557272
```

>**ref|NW_001841731.1|** G Homo sapiens unplaced genomic contig, alternate assembly HuRef
DEGEN_1103279082069, whole genome shotgun sequence
Length=4576

GENE ID: 100288203 LOC100288203 | WAS protein homolog associated with actin,
golgi membranes and microtubules pseudogene [Homo sapiens]

```
 Score =  968 bits (524),  Expect = 0.0
 Identities = 524/524 (100%), Gaps = 0/524 (0%)
 Strand=Plus/Minus

Query  1     GTTGGTCAGGCTGGTGTCGAAGTCCTGACCTCAAGTGATCCACCCGCCTCGGCCTCCCAA  60
             ||||||||||||||||||||||||||||||||||||||||||||||||||||||||||||
Sbjct  2018  GTTGGTCAGGCTGGTGTCGAAGTCCTGACCTCAAGTGATCCACCCGCCTCGGCCTCCCAA  1959

Query  61    AGTGCTGGAATTACAGGCGTGAGCCACGGTGCCTGGCCAAAAGTCTCCATATTATTAAAC  120
             ||||||||||||||||||||||||||||||||||||||||||||||||||||||||||||
Sbjct  1958  AGTGCTGGAATTACAGGCGTGAGCCACGGTGCCTGGCCAAAAGTCTCCATATTATTAAAC  1899

Query  121   AATCTTCAGAAGCACAGTGCTGAATGACTACAGTAATAATATTCTGCCATGGATATATCA  180
             ||||||||||||||||||||||||||||||||||||||||||||||||||||||||||||
Sbjct  1898  AATCTTCAGAAGCACAGTGCTGAATGACTACAGTAATAATATTCTGCCATGGATATATCA  1839

Query  181   TAATTTACTTAACAATTCTTGTTTTATTGGGCATTTTTGATGGAGGATGATGACATTTTC  240
             ||||||||||||||||||||||||||||||||||||||||||||||||||||||||||||
Sbjct  1838  TAATTTACTTAACAATTCTTGTTTTATTGGGCATTTTTGATGGAGGATGATGACATTTTC  1779

Query  241   ATATTTAATCAATATTTTAAATTGATGTATTGAAAGTTGAGAACATGAAGGTTTCTTTTG  300
             ||||||||||||||||||||||||||||||||||||||||||||||||||||||||||||
Sbjct  1778  ATATTTAATCAATATTTTAAATTGATGTATTGAAAGTTGAGAACATGAAGGTTTCTTTTG  1719

Query  301   TTTAGTTTTGTTTGTTGGGTATGTATTACACTGTCTGACTTGAGCTTTATTCACATTTGC  360
             ||||||||||||||||||||||||||||||||||||||||||||||||||||||||||||
Sbjct  1718  TTTAGTTTTGTTTGTTGGGTATGTATTACACTGTCTGACTTGAGCTTTATTCACATTTGC  1659

Query  361   TCTCTAGGTTATCCAAGGACATGGAAAAGCCAACACCATGGTAGCATTAATGAAAGTTTA  420
             ||||||||||||||||||||||||||||||||||||||||||||||||||||||||||||
Sbjct  1658  TCTCTAGGTTATCCAAGGACATGGAAAAGCCAACACCATGGTAGCATTAATGAAAGTTTA  1599

Query  421   CCAAGAGGAAGATGAAGCCTACCAGGAATTAGTTACCATGGCAACCATGTTTTTCCAGTA  480
             ||||||||||||||||||||||||||||||||||||||||||||||||||||||||||||
Sbjct  1598  CCAAGAGGAAGATGAAGCCTACCAGGAATTAGTTACCATGGCAACCATGTTTTTCCAGTA  1539
```

```
Query  481      CTTACTGCAGCCATTTAGGGCTATGCGAGAAGTTGCAACTTTAT  524
                ||||||||||||||||||||||||||||||||||||||||||||
Sbjct  1538     CTTACTGCAGCCATTTAGGGCTATGCGAGAAGTTGCAACTTTAT  1495
```

>**ref|NT_078094.2|** D Homo sapiens chromosome 15 genomic contig, GRCh37.p5 Primary Assembly
Length=868660

Sort alignments for this subject sequence by:
E value **Score** **Percent identity**
Query start position **Subject start position**

Features flanking this part of subject sequence:
142770 bp at 5' side: magnesium transporter NIPA1 isoform 2
200705 bp at 3' side: putative golgin subfamily A member 6-like protein 6-like ...

Score = 874 bits (473), Expect = 0.0
Identities = 508/525 (97%), Gaps = 1/525 (0%)
Strand=Plus/Minus

```
Query  1        GTTGGTCAGGCTGGTGTCGAAGTCCTGACCTCAAGTGATCCACCCGCCTCGGCCTCCCAA  60
                ||||||||||||||| ||||||||||||||||||||||||||||||||| ||||||||||
Sbjct  559373   GTTGGTCAGGCTGGTCTCGAAGTCCTGACCTCAAGTGATCCACCCGCCTTGGCCTCCCAA  559314

Query  61       AGTGCTGGAATTACAGGCGTGAGCCACGGTGCCTGGCCAAAAGTCTCCATATTATTAAAC  120
                |||||||||||||| ||| |||||||| ||||||||||||||||||||||||||||||||
Sbjct  559313   AGTGCTGGAATTACCGGCATGAGCCACCGTGCCTGGCCAAAAGTCTCCATATTATTAAAC  559254

Query  121      AATCTTCAGAAGCACAGTGCTGAATGACTACAGTAATAATATTCTGCCATGGATATATCA  180
                |||||||||||||||||||||||||||||||| |||||||||||||||||||||||||||
Sbjct  559253   AATCTTCAGAAGCACAGTGCTGAATGACTACACTAATAATATTCTGCCATGGATATATCA  559194

Query  181      TAATTTACTTAACAATTCTTGTTTTATTGGGCATTTTTGATGGAGGATGATGACATTTTC  240
                |||||| |||||||||||||||||||||||||||||||||||||||||||| ||||||||
Sbjct  559193   TAATTTTCTTAACAATTCTTGTTTTATTGGGCATTTTTGATGGAGGATGATAACATTTTC  559134

Query  241      ATATTTAATCAATATTTTAAATTGATGTATTGAAAGTTGAGAACATGAAGGTTTCTTTTG  300
                 |||||||||||||||||||||||||||||||||||||||||||||||||||||||||||
Sbjct  559133   GTATTTAATCAATATTTTAAATTGATGTATTGAAAGTTGAGAACATGAAGGTTTCTTTTG  559074

Query  301      TTTAGTTTTGTTTGTTGGGTATGTATTACACTGT-CTGACTTGAGCTTTATTCACATTTG  359
                ||||| |||||||||||||||||||||||||||| |||||||||||||||||||||||||
Sbjct  559073   TTTAGCTTTGTTTGTTGGGTATGTATTACACTGTCCTGACTTGAGCTTTATTCACATTTG  559014

Query  360      CTCTCTAGGTTATCCAAGGACATGGAAAAGCCAACACCATGGTAGCATTAATGAAAGTTT  419
                ||||||||||||| ||||||||  ||||||||||||||||||||||||||||||||||||
Sbjct  559013   CTCTCTAGGTTATTCAAGGACACCGAAAAGCCAACACCATGGTAGCATTAATGAAAGTTT  558954

Query  420      ACCAAGAGGAAGATGAAGCCTACCAGGAATTAGTTACCATGGCAACCATGTTTTTCCAGT  479
                |||||||||||||||||||||||||||||||||||||| ||||||||| ||| |||||||
Sbjct  558953   ACCAAGAGGAAGATGAAGCCTACCAGGAATTAGTTACCGTGGCAACCACGTTCTTCCAGT  558894

Query  480      ACTTACTGCAGCCATTTAGGGCTATGCGAGAAGTTGCAACTTTAT  524
                |||||||||||||||||||||||||||||||||||||||||||||
Sbjct  558893   ACTTACTGCAGCCATTTAGGGCTATGCGAGAAGTTGCAACTTTAT  558849
```

>**ref|NW_001838191.2|** D Homo sapiens chromosome 15 genomic contig, alternate assembly HuRef SCAF_1103279188272, whole genome shotgun sequence
Length=593693

Sort alignments for this subject sequence by:
E value **Score** **Percent identity**
Query start position **Subject start position**

Features flanking this part of subject sequence:
94434 bp at 3' side: putative golgin subfamily A member 6-like protein 6-like

Score = 874 bits (473), Expect = 0.0
Identities = 508/525 (97%), Gaps = 1/525 (0%)
Strand=Plus/Plus

```
Query  1        GTTGGTCAGGCTGGTGTCGAAGTCCTGACCTCAAGTGATCCACCCGCCTCGGCCTCCCAA  60
                ||||||||||||||| ||||||||||||||||||||||||||||||||| ||||||||||
Sbjct  121961   GTTGGTCAGGCTGGTCTCGAAGTCCTGACCTCAAGTGATCCACCCGCCTTGGCCTCCCAA  122020

Query  61       AGTGCTGGAATTACAGGCGTGAGCCACGGTGCCTGGCCAAAAGTCTCCATATTATTAAAC  120
                |||||||||||||| ||| |||||||| ||||||||||||||||||||||||||||||||
Sbjct  122021   AGTGCTGGAATTACCGGCATGAGCCACCGTGCCTGGCCAAAAGTCTCCATATTATTAAAC  122080

Query  121      AATCTTCAGAAGCACAGTGCTGAATGACTACAGTAATAATATTCTGCCATGGATATATCA  180
                |||||||||||||||||||||||||||||||| |||||||||||||||||||||||||||
Sbjct  122081   AATCTTCAGAAGCACAGTGCTGAATGACTACACTAATAATATTCTGCCATGGATATATCA  122140

Query  181      TAATTTACTTAACAATTCTTGTTTTATTGGGCATTTTTGATGGAGGATGATGACATTTTC  240
                |||||| |||||||||||||||||||||||||||||||||||||||||||| ||||||||
Sbjct  122141   TAATTTTCTTAACAATTCTTGTTTTATTGGGCATTTTTGATGGAGGATGATAACATTTTC  122200

Query  241      ATATTTAATCAATATTTTAAATTGATGTATTGAAAGTTGAGAACATGAAGGTTTCTTTTG  300
                 |||||||||||||||||||||||||||||||||||||||||||||||||||||||||||
Sbjct  122201   GTATTTAATCAATATTTTAAATTGATGTATTGAAAGTTGAGAACATGAAGGTTTCTTTTG  122260

Query  301      TTTAGTTTTGTTTGTTGGGTATGTATTACACTGT-CTGACTTGAGCTTTATTCACATTTG  359
                ||||| |||||||||||||||||||||||||||| |||||||||||||||||||||||||
Sbjct  122261   TTTAGCTTTGTTTGTTGGGTATGTATTACACTGTCCTGACTTGAGCTTTATTCACATTTG  122320

Query  360      CTCTCTAGGTTATCCAAGGACATGGAAAAGCCAACACCATGGTAGCATTAATGAAAGTTT  419
                ||||||||||||| ||||||||  ||||||||||||||||||||||||||||||||||||
Sbjct  122321   CTCTCTAGGTTATTCAAGGACACCGAAAAGCCAACACCATGGTAGCATTAATGAAAGTTT  122380

Query  420      ACCAAGAGGAAGATGAAGCCTACCAGGAATTAGTTACCATGGCAACCATGTTTTTCCAGT  479
```



```
                |||||||||||||||||||||||||||||||||||||| ||||||||| ||| |||||||
Sbjct  122381   ACCAAGAGGAAGATGAAGCCTACCAGGAATTAGTTACCGTGGCAACCACGTTCTTCCAGT  122440

Query  480      ACTTACTGCAGCCATTTAGGGCTATGCGAGAAGTTGCAACTTTAT  524
                |||||||||||||||||||||||||||||||||||||||||||||
Sbjct  122441   ACTTACTGCAGCCATTTAGGGCTATGCGAGAAGTTGCAACTTTAT  122485
```

>**ref|NT_026446.14|** D Homo sapiens chromosome 15 genomic contig, GRCh37.p5 Primary Assembly
Length=5594590

Sort alignments for this subject sequence by:
E value **Score** **Percent identity**
Query start position **Subject start position**

Features flanking this part of subject sequence:
32762 bp at 5' side: putative golgin subfamily A member 8I

Score = 863 bits (467), Expect = 0.0
Identities = 506/525 (96%), Gaps = 1/525 (0%)
Strand=Plus/Plus

```
Query  1        GTTGGTCAGGCTGGTGTCGAAGTCCTGACCTCAAGTGATCCACCCGCCTCGGCCTCCCAA  60
                ||||||||||||||| ||||||||||||||||||||||||||||||||||||||||||||
Sbjct  5421035  GTTGGTCAGGCTGGTCTCGAAGTCCTGACCTCAAGTGATCCACCCGCCTCGGCCTCCCAA  5421094

Query  61       AGTGCTGGAATTACAGGCGTGAGCCACGGTGCCTGGCCAAAAGTCTCCATATTATTAAAC  120
                |||||||||||||| ||| |||||||| ||||||||||||||||||||||||||||||||
Sbjct  5421095  AGTGCTGGAATTACCGGCATGAGCCACCGTGCCTGGCCAAAAGTCTCCATATTATTAAAC  5421154

Query  121      AATCTTCAGAAGCACAGTGCTGAATGACTACAGTAATAATATTCTGCCATGGATATATCA  180
                |||||||||||||||||||||||||||||||| |||||||||||||||||||||||||||
Sbjct  5421155  AATCTTCAGAAGCACAGTGCTGAATGACTACACTAATAATATTCTGCCATGGATATATCA  5421214

Query  181      TAATTTACTTAACAATTCTTGTTTTATTGGGCATTTTTGATGGAGGATGATGACATTTTC  240
                |||||| |||||||||||||||||||||||||||||||||||||| ||||| ||||||||
Sbjct  5421215  TAATTTTCTTAACAATTCTTGTTTTATTGGGCATTTTTGATGGAGAATGATAACATTTTC  5421274

Query  241      ATATTTAATCAATATTTTAAATTGATGTATTGAAAGTTGAGAACATGAAGGTTTCTTTTG  300
                 ||||||||||||||||||||||||||||||||||||||||||||||||||||||||| |
Sbjct  5421275  GTATTTAATCAATATTTTAAATTGATGTATTGAAAGTTGAGAACATGAAGGTTTCTTTCG  5421334

Query  301      TTTAGTTTTGTTTGTTGGGTATGTATTACACTGT-CTGACTTGAGCTTTATTCACATTTG  359
                ||||| |||||||||||||||||||||||||||| |||||||||||||||||||||||||
Sbjct  5421335  TTTAGCTTTGTTTGTTGGGTATGTATTACACTGTCCTGACTTGAGCTTTATTCACATTTG  5421394

Query  360      CTCTCTAGGTTATCCAAGGACATGGAAAAGCCAACACCATGGTAGCATTAATGAAAGTTT  419
                ||||||||||||| |||||||| |||||||||||||||||||||||||||||||||||||
Sbjct  5421395  CTCTCTAGGTTATTCAAGGACACGGAAAAGCCAACACCATGGTAGCATTAATGAAAGTTT  5421454

Query  420      ACCAAGAGGAAGATGAAGCCTACCAGGAATTAGTTACCATGGCAACCATGTTTTTCCAGT  479
                |||||||||||||||||||||||||||||||||||||| ||||||||| ||| |||||||
Sbjct  5421455  ACCAAGAGGAAGATGAAGCCTACCAGGAATTAGTTACCGTGGCAACCACGTTCTTCCAGT  5421514

Query  480      ACTTACTGCAGCCATTTAGGGCTATGCGAGAAGTTGCAACTTTAT  524
                ||||| ||| |||||||||||||||||||||||||||||||||||
Sbjct  5421515  ACTTATTGCGGCCATTTAGGGCTATGCGAGAAGTTGCAACTTTAT  5421559
```

>**ref|NW_001838221.1|** D Homo sapiens chromosome 15 genomic contig, alternate assembly HuRef SCAF_1103279188173, whole genome shotgun sequence
Length=1688394

Sort alignments for this subject sequence by:
E value **Score** **Percent identity**
Query start position **Subject start position**

Features in this part of subject sequence:
WASP homolog-associated protein with actin, membranes and...

Score = 706 bits (382), Expect = 0.0
Identities = 452/485 (93%), Gaps = 7/485 (1%)
Strand=Plus/Plus

```
Query  46       GCCTCGGCCTCCCAAAG-TGCTGGAATTACAGGCGTGAGCCACGGTGCCTGGCCAAAAGT  104
                ||||| |||||||  ||  ||||| |||||||| |||||||||  |||||||||||||||
Sbjct  291590   GCCTCAGCCTCCC-CAGCAGCTGGGATTACAGGTGTGAGCCACCATGCCTGGCCAAAAGT  291648

Query  105      CTCCATATTATTAAACAATCTTCAGAAGCACAGTGCTGAATGACTACAGTAATAATATTC  164
                ||||||||||||||||||||||||||||||||||||||||||||||||||||||||||||
Sbjct  291649   CTCCATATTATTAAACAATCTTCAGAAGCACAGTGCTGAATGACTACAGTAATAATATTC  291708

Query  165      TGCCATGGATATATCATAATTTACTTAACAATTCTTGTTTTATTGGGCATTTTTGATGGA  224
                |  ||||||||||||||| ||| |||||| ||||||||||||||||||||||||||||||
Sbjct  291709   TTACATGGATATATCATACTTTTCTTAACCATTCTTGTTTTATTGGGCATTTTTGATGGA  291768

Query  225      GGATGATGACATTTTCATATTTAATCAATATTTTAAATTGATGTATTGAAAGTTGAGAAC  284
                ||||||| ||||||||||||||||||| ||||| ||||||||||||| ||||||||||||
Sbjct  291769   GGATGATAACATTTTCATATTTAATCAGTATTTAAAATTGATGTATTAAAAGTTGAGAAC  291828

Query  285      ATGAAGGTTTCTTTTGTTTAGTTTTGTTTGTTGGGTATGTATTACA----CTGTC-TGAC  339
                |||||||||||||||||||||||||||||||||||||||||||| |    ||||| ||||
Sbjct  291829   ATGAAGGTTTCTTTTGTTTAGTTTTGTTTGTTGGGTATGTATTAAATATACTGTCCTGAC  291888

Query  340      TTGAGCTTTATTCACATTTGCTCTCTAGGTTATCCAAGGACATGGAAAAGCCAACACCAT  399
                |||||||||||||||||||||| |||||||||| |||||||| |||||||||||||||||
Sbjct  291889   TTGAGCTTTATTCACATTTGCTTTCTAGGTTATTCAAGGACACGGAAAAGCCAACACCAT  291948

Query  400      GGTAGCATTAATGAAAGTTTACCAAGAGGAAGATGAAGCCTACCAGGAATTAGTTACCAT  459
                ||||||||||||||| ||||||||||||||||||||||| ||||||||||| |||||| |
Sbjct  291949   GGTAGCATTAATGAACGTTTACCAAGAGGAAGATGAAGCATACCAGGAATTGGTTACCGT  292008
```

```
Query  460     GGCAACCATGTTTTTCCAGTACTTACTGCAGCCATTTAGGGCTATGCGAGAAGTTGCAAC  519
               |||||||||||| ||||||||||| |||||||||||||||||||||||||||||||||||
Sbjct  292009  GGCAACCATGTTCTTCCAGTACTTATTGCAGCCATTTAGGGCTATGCGAGAAGTTGCAAC  292068

Query  520     TTTAT  524
               |||||
Sbjct  292069  TTTAT  292073
```

>ref|NT_077661.3| Homo sapiens chromosome 15 genomic contig, GRCh37.p5 Primary Assembly
Length=2104828

Sort alignments for this subject sequence by:
E value **Score** **Percent identity**
Query start position **Subject start position**

Features in this part of subject sequence:
WASP homolog-associated protein with actin, membranes and...

Score = 701 bits (379), Expect = 0.0
Identities = 451/485 (93%), Gaps = 7/485 (1%)
Strand=Plus/Plus

```
Query  46      GCCTCGGCCTCCCAAAG-TGCTGGAATTACAGGCGTGAGCCACGGTGCCTGGCCAAAAGT  104
               ||||| |||||||  ||  ||||| |||||||| |||||||||  ||||||| |||||||
Sbjct  601883  GCCTCAGCCTCCC-CAGCAGCTGGGATTACAGGTGTGAGCCACCATGCCTGGACAAAAGT  601941

Query  105     CTCCATATTATTAAACAATCTTCAGAAGCACAGTGCTGAATGACTACAGTAATAATATTC  164
               ||||||||||||||||||||||||||||||||||||||||||||||||||||||||||||
Sbjct  601942  CTCCATATTATTAAACAATCTTCAGAAGCACAGTGCTGAATGACTACAGTAATAATATTC  602001

Query  165     TGCCATGGATATATCATAATTTACTTAACAATTCTTGTTTTATTGGGCATTTTTGATGGA  224
               |  ||||||||||||||| ||| |||||| ||||||||||||||||||||||||||||||
Sbjct  602002  TTACATGGATATATCATACTTTTCTTAACCATTCTTGTTTTATTGGGCATTTTTGATGGA  602061

Query  225     GGATGATGACATTTTCATATTTAATCAATATTTTAAATTGATGTATTGAAAGTTGAGAAC  284
               ||||||| ||||||||||||||||||| ||||| ||||||||||||| ||||||||||||
Sbjct  602062  GGATGATAACATTTTCATATTTAATCAGTATTTAAAATTGATGTATTAAAAGTTGAGAAC  602121

Query  285     ATGAAGGTTTCTTTTGTTTAGTTTTGTTTGTTGGGTATGTATTACA----CTGTC-TGAC  339
               |||||||||||||||||||||||||||||||||||||||||||| |    ||||| ||||
Sbjct  602122  ATGAAGGTTTCTTTTGTTTAGTTTTGTTTGTTGGGTATGTATTAAATATACTGTCCTGAC  602181

Query  340     TTGAGCTTTATTCACATTTGCTCTCTAGGTTATCCAAGGACATGGAAAAGCCAACACCAT  399
               |||||||||||||||||||||| |||||||||| |||||||| |||||||||||||||||
Sbjct  602182  TTGAGCTTTATTCACATTTGCTTTCTAGGTTATTCAAGGACACGGAAAAGCCAACACCAT  602241

Query  400     GGTAGCATTAATGAAAGTTTACCAAGAGGAAGATGAAGCCTACCAGGAATTAGTTACCAT  459
               ||||||||||||||| ||||||||||||||||||||||| ||||||||||| |||||| |
Sbjct  602242  GGTAGCATTAATGAACGTTTACCAAGAGGAAGATGAAGCATACCAGGAATTGGTTACCGT  602301

Query  460     GGCAACCATGTTTTTCCAGTACTTACTGCAGCCATTTAGGGCTATGCGAGAAGTTGCAAC  519
               |||||||||||| ||||||||||| |||||||||||||||||||||||||||||||||||
Sbjct  602302  GGCAACCATGTTCTTCCAGTACTTATTGCAGCCATTTAGGGCTATGCGAGAAGTTGCAAC  602361

Query  520     TTTAT  524
               |||||
Sbjct  602362  TTTAT  602366
```

>ref|NT_011362.10| Homo sapiens chromosome 20 genomic contig, GRCh37.p5 Primary Assembly
Length=31409461

Features flanking this part of subject sequence:
24374 bp at 5' side: engulfment and cell motility protein 2
82185 bp at 3' side: zinc finger protein 334 isoform a

Score = 396 bits (214), Expect = 1e-108
Identities = 242/256 (95%), Gaps = 0/256 (0%)
Strand=Plus/Minus

```
Query  1330      AGATTTATTTTCTTTTTGAATATATTTTCTACACCATGGTTTCTCAACCAGAGGTGATAC  1389
                 ||||||||||||| ||||||||||||||||||||||||||||||||||||||||||||||
Sbjct  15243842  AGATTTATTTTCTCTTTGAATATATTTTCTACACCATGGTTTCTCAACCAGAGGTGATAC  15243783

Query  1390      AGCACTGTCTCCAAATGTGGGGCTGTTGTGTATTGTCACGGGGAGGAGAAATGCTACTGG  1449
                 |||||   ||||||||||||||||| ||||||||||||||||||||||||||||||||||
Sbjct  15243782  AGCACCCCCTCCAAATGTGGGGCTGCTGTGTATTGTCACGGGGAGGAGAAATGCTACTGG  15243723

Query  1450      CATTTAGTGGATGATGGTCAAGGACACTAAGCAACCTGCAATGTGTGGATCAATCTTACA  1509
                 |||||| |||||||||||||||||||||||| |||||||||||||||||||| |||||||
Sbjct  15243722  CATTTAATGGATGATGGTCAAGGACACTAAGTAACCTGCAATGTGTGGATCAGTCTTACA  15243663

Query  1510      CAACGAAGGACTGTTCCACCCCAAATGCCAATGGTACCCTGTTGAGAAAGGTTAGTCTAT  1569
                 |||  ||| | ||||||||||||||||||| ||||||||||||||||||||||| |||||
Sbjct  15243662  CAATAAAGAATTGTTCCACCCCAAATGCCAGTGGTACCCTGTTGAGAAAGGTTACTCTAT  15243603

Query  1570      GTGGACAGTGAATTAT  1585
                 ||||||||||||||||
Sbjct  15243602  GTGGACAGTGAATTAT  15243587
```

>ref|NW_001838666.1| Homo sapiens chromosome 20 genomic contig, alternate assembly HuRef SCAF_1103279188360, whole genome shotgun sequence
Length=19874876

Features flanking this part of subject sequence:
24384 bp at 5' side: engulfment and cell motility protein 2
82196 bp at 3' side: zinc finger protein 334 isoform a

Score = 396 bits (214), Expect = 1e-108
Identities = 242/256 (95%), Gaps = 0/256 (0%)
Strand=Plus/Minus

```
Query  1330     AGATTTATTTTCTTTTTGAATATATTTTCTACACCATGGTTTCTCAACCAGAGGTGATAC  1389
                ||||||||||||| ||||||||||||||||||||||||||||||||||||||||||||||
Sbjct  8109497  AGATTTATTTTCTCTTTGAATATATTTTCTACACCATGGTTTCTCAACCAGAGGTGATAC  8109438

Query  1390     AGCACTGTCTCCAAATGTGGGGCTGTTGTGTATTGTCACGGGGAGGAGAAATGCTACTGG  1449
                |||||   ||||||||||||||||| ||||||||||||||||||||||||||||||||||
Sbjct  8109437  AGCACCCCCTCCAAATGTGGGGCTGCTGTGTATTGTCACGGGGAGGAGAAATGCTACTGG  8109378

Query  1450     CATTTAGTGGATGATGGTCAAGGACACTAAGCAACCTGCAATGTGTGGATCAATCTTACA  1509
                |||||| |||||||||||||||||||||||| |||||||||||||||||||| |||||||
Sbjct  8109377  CATTTAATGGATGATGGTCAAGGACACTAAGTAACCTGCAATGTGTGGATCAGTCTTACA  8109318

Query  1510     CAACGAAGGACTGTTCCACCCCAAATGCCAATGGTACCCTGTTGAGAAAGGTTAGTCTAT  1569
                |||  ||| | ||||||||||||||||||| ||||||||||||||||||||||| |||||
Sbjct  8109317  CAATAAAGAATTGTTCCACCCCAAATGCCAGTGGTACCCTGTTGAGAAAGGTTACTCTAT  8109258

Query  1570     GTGGACAGTGAATTAT  1585
                ||||||||||||||||
Sbjct  8109257  GTGGACAGTGAATTAT  8109242
```

>ref|NW_001838205.1| Homo sapiens chromosome 15 genomic contig, alternate assembly HuRef SCAF_1103279183344, whole genome shotgun sequence
Length=8319

GENE ID: 100288615 LOC100288615 | WAS protein homolog associated with actin, golgi membranes and microtubules pseudogene [Homo sapiens]
(10 or fewer PubMed links)

Sort alignments for this subject sequence by:
E value **Score** **Percent identity**
Query start position **Subject start position**

Score = 320 bits (173), Expect = 8e-86
Identities = 173/173 (100%), Gaps = 0/173 (0%)
Strand=Plus/Plus

```
Query  678   CAGGAATGCAAAAAGAAATGGAACAGGATGTGAAGAGATTTGGCCAGGCTGCCTGGGCCA  737
             ||||||||||||||||||||||||||||||||||||||||||||||||||||||||||||
Sbjct  3804  CAGGAATGCAAAAAGAAATGGAACAGGATGTGAAGAGATTTGGCCAGGCTGCCTGGGCCA  3863

Query  738   CAGCAATTCCCAGGTTGGAAAAACTTAAGCTAATGCTAGCTCAAGAGACTCTGCAACTCA  797
             ||||||||||||||||||||||||||||||||||||||||||||||||||||||||||||
Sbjct  3864  CAGCAATTCCCAGGTTGGAAAAACTTAAGCTAATGCTAGCTCAAGAGACTCTGCAACTCA  3923

Query  798   TGAGAGCGAAAGAATTGTATTTAAATCGCAAAAGAGCTGAAATTCAGGGAAAG  850
             |||||||||||||||||||||||||||||||||||||||||||||||||||||
Sbjct  3924  TGAGAGCGAAAGAATTGTATTTAAATCGCAAAAGAGCTGAAATTCAGGGAAAG  3976
```
