# Supplementary material for: Tissue-based Alzheimer gene expression markers–comparison of multiple machine learning approaches and investigation of redundancy in small biomarker sets
Source: BMC Bioinformatics. 2012 Oct 15;13:266. doi: 10.1186/1471-2105-13-266 (PMC3574043; doi:10.1186/1471-2105-13-266)

Supplement: Comparison of Results

In our paper we refer to an analysis of the pluripotency data presented in [1], where we found an error in the preprocessing step; the correlation structure of the samples was not adequately factored in for the three-fold cross-validation leading to slightly elevated classification accuracies. For this reason, we redid all calculations based on a correct partitioning of the PLURI data set into three subsets (see Methods). This file shows the classification accuracies obtained for the PLURI data set using the incorrect and the corrected partitions as well as the accuracies for the AD data set.

[1] L. Scheubert, R. Schmidt, D. Repsilber, M. Lustrek, and G. Fuellen. Learning biomarkers of pluripotent stem cells in mouse. *DNA Res.*, 18:233–251, 2011.

Classification accuracy of six classifiers (cf. Table 1)

The classification results of six different methods obtained on the three 1,000 gene data sets PLURI (incorrect partitioning), PLURI and AD. The classification accuracy is computed as average from a 3-fold cross-validation.

	PLURI (incorrect partitioning)	PLURI	AD
Naive Bayes	94.9%	87.1%	81.4%
C4.5 decision tree	93.4%	95.1%	78.9%
Nearest neighbor	100%	96.5%	87.0%
Random Forest	97.6%	97.2%	87.0%
SVM + Gaussian kernel	100%	97.9%	85.7%
SVM + linear kernel	100%	99.0%	91.9%

Classification accuracy of the selected genes (cf. Figure 1)

Classification accuracy of three classifiers using incrementally smaller sets of genes, identified by our three feature selection methods.

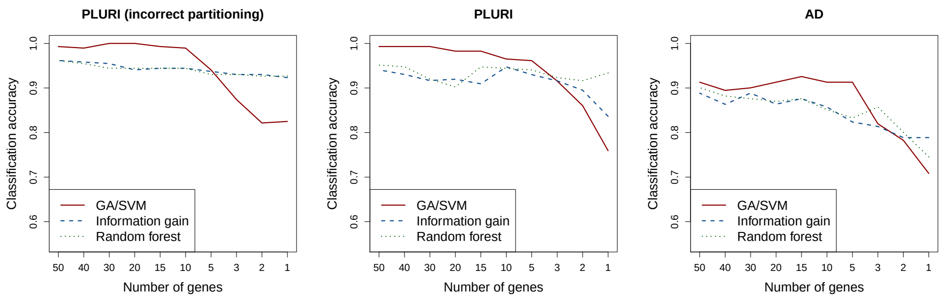

Abbildung 1: Classifier: SVM with linear kernel

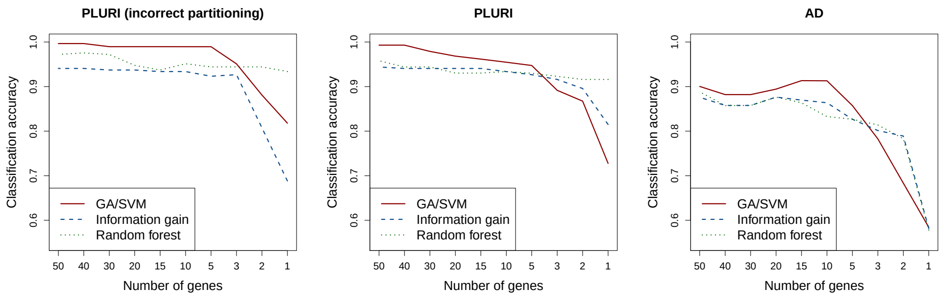

Abbildung 2: Classifier: SVM with Gaussian kernel

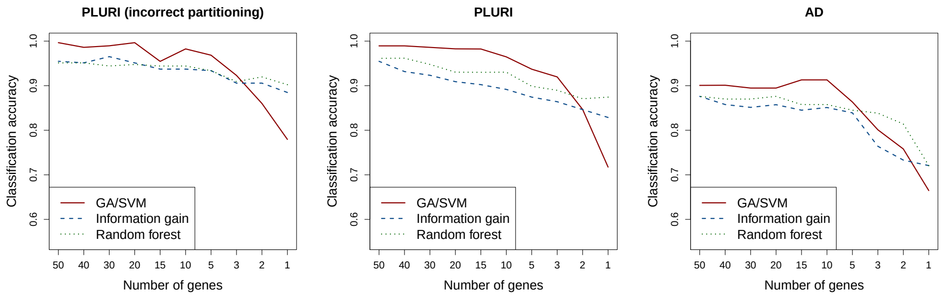

Abbildung 3: Classifier: Random forest

Classification accuracy of the selected genes (cf. Figure 3)

Classification accuracy measured by an SVM with Gaussian kernel. For training the classifiers we use incrementally smaller sets of best ranked genes (combined list) and the small gene sets found by the GA/SVM in single runs.

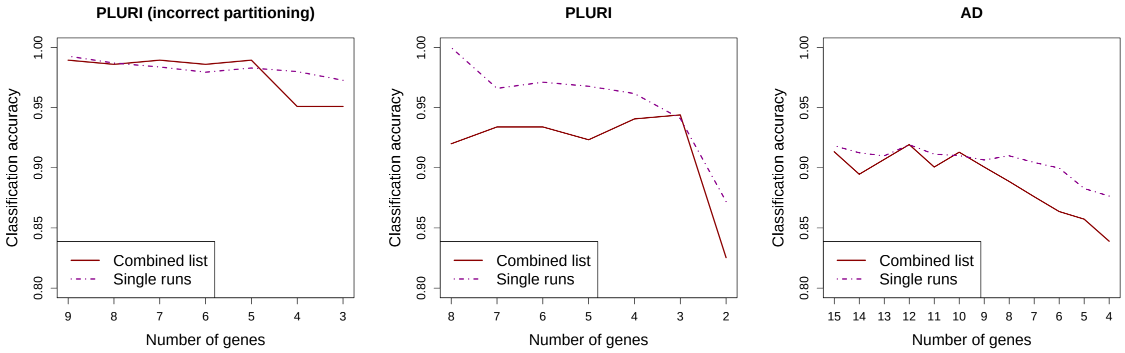

Supplement: Additional file 2 — Comparison of the results obtained on the PLURI data set without the correct partitioning, the PLURI data set and the AD data set. [file 1471-2105-13-266-S2.pdf]
