# Supplementary material for: Tissue-based Alzheimer gene expression markers–comparison of multiple machine learning approaches and investigation of redundancy in small biomarker sets
Source: BMC Bioinformatics. 2012 Oct 15;13:266. doi: 10.1186/1471-2105-13-266 (PMC3574043; doi:10.1186/1471-2105-13-266)
Supplement: Additional file 3 — Partitioning of the AD data set. [file 1471-2105-13-266-S3.pdf]

AD partitioning

AD data set: Partitioning of the GEO data sets into three subsets (folds).

Fold 1	Fold 2	Fold 3
GSM119617	GSM119615	GSM119619
GSM119618	GSM119616	GSM119620
GSM119621	GSM119622	GSM119623
GSM119626	GSM119624	GSM119625
GSM119627	GSM119628	GSM119631
GSM119629	GSM119632	GSM119633
GSM119630	GSM119635	GSM119636
GSM119634	GSM119637	GSM119638
GSM119639	GSM119641	GSM119643
GSM119640	GSM119644	GSM119645
GSM119642	GSM119646	GSM119647
GSM119651	GSM119648	GSM119649
GSM119652	GSM119653	GSM119650
GSM119654	GSM119656	GSM119655
GSM119658	GSM119659	GSM119657
GSM119664	GSM119661	GSM119660
GSM119665	GSM119667	GSM119662
GSM119673	GSM119671	GSM119663
GSM119675	GSM119674	GSM119666
GSM119676	GSM119677	GSM119668
GSM119678	GSM119679	GSM119669
GSM119681	GSM119682	GSM119670
GSM119687	GSM119684	GSM119672
GSM119688	GSM238790	GSM119680
GSM238791	GSM238797	GSM119683
GSM238794	GSM238798	GSM119685
GSM238795	GSM238801	GSM119686
GSM238799	GSM238803	GSM238763
GSM238802	GSM238804	GSM238792
GSM238807	GSM238806	GSM238793
GSM238808	GSM238812	GSM238796
GSM238810	GSM238815	GSM238800
GSM238811	GSM238817	GSM238805
GSM238813	GSM238820	GSM238809
GSM238816	GSM238823	GSM238821

Continued on next page

Fold 1	Fold 2	Fold 3
GSM238818	GSM238835	GSM238822
GSM238819	GSM238839	GSM238825
GSM238824	GSM238841	GSM238826
GSM238827	GSM238845	GSM238838
GSM238834	GSM238847	GSM238840
GSM238837	GSM238851	GSM238842
GSM238843	GSM238855	GSM238856
GSM238844	GSM238857	GSM238858
GSM238846	GSM238860	GSM238861
GSM238848	GSM238865	GSM238863
GSM238854	GSM238867	GSM238864
GSM238862	GSM238874	GSM238868
GSM238870	GSM238877	GSM238871
GSM238873	GSM238942	GSM238872
GSM238875	GSM238944	GSM238948
GSM238941	GSM238945	GSM238949
GSM238943	GSM238947	GSM238951
GSM238946	GSM238952	GSM238953
GSM238955		GSM238963
