# Supplementary material for: Tissue-based Alzheimer gene expression markers–comparison of multiple machine learning approaches and investigation of redundancy in small biomarker sets
Source: BMC Bioinformatics. 2012 Oct 15;13:266. doi: 10.1186/1471-2105-13-266 (PMC3574043; doi:10.1186/1471-2105-13-266)
Supplement: Additional file 4 — Partitioning of the PLURI data set. [file 1471-2105-13-266-S4.pdf]

PLURI partitioning

PLURI data set: Partitioning of the GEO data sets into three subsets (folds).

Fold 1	Fold 2	Fold 3
GSM144634	GSM144622	GSM144635
GSM185509	GSM144623	GSM198065
GSM185511	GSM144624	GSM234773
GSM185513	GSM144636	GSM241851
GSM198062	GSM185510	GSM241852
GSM198064	GSM185512	GSM241856
GSM198066	GSM198063	GSM241858
GSM198070	GSM198067	GSM241863
GSM241853	GSM198072	GSM241865
GSM241857	GSM234772	GSM241869
GSM241859	GSM241847	GSM241873
GSM241860	GSM241848	GSM241876
GSM241861	GSM241849	GSM252064
GSM241862	GSM241850	GSM252068
GSM241864	GSM241854	GSM252069
GSM241868	GSM241855	GSM252072
GSM241870	GSM241866	GSM252073
GSM241872	GSM241867	GSM252077
GSM241874	GSM241871	GSM252082
GSM241875	GSM252066	GSM252092
GSM252065	GSM252067	GSM252093
GSM252070	GSM252075	GSM252094
GSM252071	GSM252076	GSM252095
GSM252074	GSM252078	GSM252104
GSM252080	GSM252079	GSM252107
GSM252086	GSM252081	GSM252109
GSM252087	GSM252083	GSM252112
GSM252090	GSM252084	GSM252115
GSM252096	GSM252085	GSM252116
GSM252098	GSM252088	GSM252125
GSM252103	GSM252089	GSM252129
GSM252106	GSM252091	GSM252133
GSM252110	GSM252097	GSM265042
GSM252114	GSM252099	GSM272035
GSM252117	GSM252100	GSM272036

Continued on next page

Fold 1	Fold 2	Fold 3
GSM252118	GSM252101	GSM272753
GSM252120	GSM252102	GSM272839
GSM252121	GSM252105	GSM272847
GSM252122	GSM252108	GSM272890
GSM252127	GSM252111	GSM275554
GSM252130	GSM252113	GSM275555
GSM265040	GSM252119	GSM275556
GSM267415	GSM252123	GSM275557
GSM272037	GSM252124	GSM275564
GSM272052	GSM252126	GSM275567
GSM272053	GSM252128	GSM277761
GSM272054	GSM252131	GSM277765
GSM272846	GSM252132	GSM277768
GSM275544	GSM266065	GSM279200
GSM275546	GSM266837	GSM284790
GSM275551	GSM267413	GSM284793
GSM275552	GSM272836	GSM284794
GSM275558	GSM272837	GSM284797
GSM275560	GSM272848	GSM284806
GSM275562	GSM275547	GSM314040
GSM275563	GSM275548	GSM314042
GSM275581	GSM275561	GSM314043
GSM277759	GSM275566	GSM314044
GSM277760	GSM275580	GSM314046
GSM277763	GSM277757	GSM314048
GSM277764	GSM277758	GSM338371
GSM277766	GSM277762	GSM347150
GSM284788	GSM277767	GSM381301
GSM284791	GSM279201	GSM381304
GSM284795	GSM279202	GSM381305
GSM284796	GSM284789	GSM472235
GSM284799	GSM284792	GSM515594
GSM284801	GSM284798	GSM537474
GSM284805	GSM284800	GSM537475
GSM314039	GSM284807	GSM537477
GSM314045	GSM314038	GSM537478
GSM314047	GSM338369	GSM537481
GSM378798	GSM338373	GSM537488
GSM381308	GSM347151	GSM537489

Continued on next page

Fold 1	Fold 2	Fold 3
GSM446646	GSM378796	GSM537490
GSM472236	GSM378797	GSM85006
GSM537476	GSM381306	GSM85008
GSM537479	GSM381307	GSM85009
GSM537482	GSM446645	GSM94859
GSM537484	GSM472237	GSM98548
GSM537485	GSM537473	GSM98550
GSM537486	GSM537480	GSM98553
GSM94856	GSM537483	GSM98554
GSM94857	GSM537487	GSM98555
GSM94858	GSM85007	GSM98556
GSM98549	GSM94860	GSM98558
GSM98551	GSM98557	GSM98561
GSM98552	GSM98559	GSM98564
GSM98560	GSM98563	GSM98569
GSM98562	GSM98566	GSM98570
GSM98565	GSM98567	GSM98573
GSM98568	GSM98571	GSM98575
GSM98572	GSM98578	GSM98576
GSM98574	GSM98579	GSM98577
GSM98582	GSM98580	GSM98581
GSM98583		
